# A Combined MS/MS and IMS Study Into the Fragmentation Pathway of Nifedipine

**DOI:** 10.1002/jms.70071

**Published:** 2026-06-11

**Authors:** Peiliang Han, Newton Thomassen, Maarten Honing

**Affiliations:** ^1^ Maastricht Multi Modal Molecular imaging (M4i) Institute, Division of Imaging Mass Spectrometry Maastricht University Maastricht the Netherlands; ^2^ Aachen Maastricht Institute Biobased Materials (AMIBM), Brightlands Chemelot Campus Geleen the Netherlands

**Keywords:** fragmentation mechanisms, ion mobility, molecular structure elucidation, MS/MS, nifedipine, ortho effect

## Abstract

Collision‐induced dissociation (CID) of small molecules, typically with a molecular mass below 1500 Da, is widely applied for the structural identification of drug metabolites, synthetic by‐products and unknown compounds. Nonetheless, the interpretation of MS/MS spectra derived from protonated or sodiated molecules mainly relies on the comparison with literature data and proposed structures usually lack rigorous mechanistic justification and conclusive evidence. The commonly applied even‐electron rule, as implemented in most literature and prediction software, does not support radical loss from protonated molecules. Similarly, fragmentation pathways of sodium adduct ions remain insufficiently explored and lack mechanistic rationalization. To improve the understanding of radical loss from even‐electron nitro‐containing compounds, a mechanistic study of fragmentation patterns of nifedipine, *m*‐nifedipine, and related analogues was performed. Fragment ions generated from [M+H]^+^ and [M+Na]^+^ of nifedipine revealed distinct mechanisms compared with its positional isomers, while showing similarities with analogues like nisoldipine and aranidipine. The nitro group at the ortho position significantly influences fragment stability, leading to a unique fragmentation mechanism described as ortho effect. The mechanism was further investigated using isotope labelled analogs, ion mobility spectrometry (IMS), precursor ion scan (PIS), and density functional theory (DFT) calculations. Different approaches in combining IMS with MS/MS demonstrated strong capability for elucidation of fragmentation pathways.

## 1 Introduction

For the structural assessment of synthetic by‐products, drug metabolites, and unknown compounds, various analytical techniques like nuclear magnetic resonance (NMR), infrared spectroscopy (IR), and mass spectrometry (MS) are commonly employed. Among these, hyphenated techniques combining gas or liquid chromatography (GC/LC) with high resolution mass spectrometry (HRMS) can be regarded as the method of choice. These approaches enable the detection of analytes at low concentration, even within complex mixtures. Together with the high resolving power and applicability to a broad range of molecules, these technologies have significantly advanced research in biology and pharmaceutical development [1, 2, 3, 4, 5].

While HRMS provides an accurate estimation of element composition, it does not provide direct evidence of three‐dimensional molecular structures. In contrast, tandem mass spectrometry (MS/MS), through characteristic fragmentation pathways following collisional activation, and ion mobility spectrometry (IMS), through collisional cross‐section and arrival time distribution (ATD) measurements, offer valuable insights into molecular constitution, configuration, and even conformation [6]. Despite the existence of well‐developed MS/MS databases for classes including glycans, lipids, and peptides [7, 8, 9, 10], the elucidation of MS/MS fragmentation pathways and the annotation of the fragment ions often rely on stable isotope labeling, literature comparison, or general fragmentation rules. These include the preferential loss of small neutral molecules with low heats of formation like H_2_O, MeOH, CH_2_O, and C_3_H_6_ [11].

In many cases, particularly for small molecules, unexpected fragmentation pathways involving bond rearrangements and radical losses are observed [12, 13, 14, 15, 16]. For example, molecules containing methoxy groups, halogen atoms, or nitro/nitroso groups are expected to generate radical loss from even‐electron ions [15, 17, 18, 19, 20]. Next, fragmentation pathways of structural isomers may show significantly different fragmentation behavior, as activation energies for analogous pathways can vary substantially [21]. In 2021, Niessen summarized the loss of even neutrals or radicals from nitro‐ and halogen‐containing molecules [20]. Nifedipine, commonly used in the treatment of Prinzmetal angina, has shown different fragmentation behavior compared with nimodipine but shared the similarity with derivatives like nisoldipine and aranidipine, likely due to the steric effect of the nitro group. This phenomenon is known as the ortho effect, which has been reported for molecules such as hydroxyphenyl carbaldehydes [22] and *N*‐alkyl*‐o‐*nitroanilines [23]. Although the fragments of nifedipine have been reported, the structures of the fragment ions proposed were not investigated in detail [20, 24, 25, 26].

**FIGURE 1:**
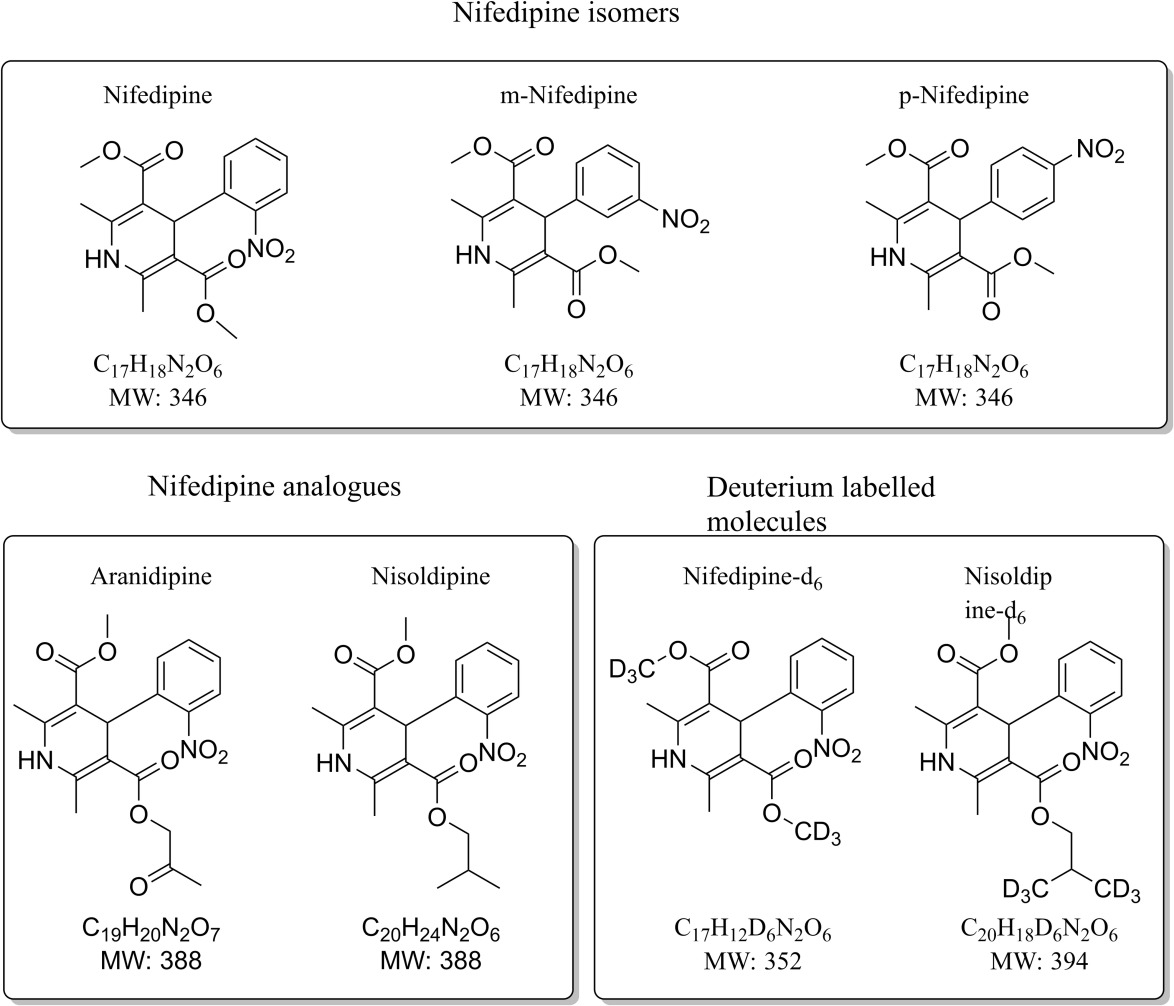
Molecular structures of analytes with labelled chemical formula and molecular weight (MW). (1) Nifedipine isomers: nifedipine, *m*‐nifedipine, and dimethyl 2,6‐dimethyl‐4‐(4‐nitrophenyl)‐1,4‐dihydropyridine‐3,5‐dicarboxylate (*p*‐nifedipine). (2) Nifedipine analogues: aranidipine and nisoldipine. (3) Deuterium labelled analogues: nifedipine‐*d*_6_ and nisoldipine‐*d*_6_.

Inspired by previous reports, the present study provides a detailed investigation of collision‐induced dissociation (CID) fragmentation pathways and ion structures, with particular attention to the ortho effect. The study includes a series of nifedipine structural analogues and isotope‐labelled compounds (Figure 1), combined with MS/MS analysis, ion mobility separation experiments, and density functional theory (DFT) calculations.

Besides the protonated molecules [M+H]^+^, sodium adducts [M+Na]^+^ were collisionally activated at varying collision energies. In addition, IMS was employed to distinguish isomeric fragment ions with different configurations [27, 28]. Particularly in the pharmaceutical industry, MS/MS coupled with IMS provides an additional analytical dimension for molecular structure elucidation, separation as well as quality control [29, 30]. In contrast to conventional IMS applications focused on isomer separation, here, this study utilizes IMS to prove the relationships among precursor ions, primary and secondary fragments through their ATD. Furthermore, key fragmentation pathways were examined in detail using precursor ion scanning (PIS).

## 2 Experimental

### 2.1 Chemicals and Sample Preparation

Nifedipine was obtained from Sigma‐Aldrich (Zwijndrecht, the Netherlands). *m*‐Nifedipine, aranidipine, and dimethyl 2,6‐dimethyl‐4‐(4‐nitrophenyl)‐1,4‐dihydropyridine‐3,5‐dicarboxylate (*p*‐nifedipine) were obtained from AmBeed (Arlington Heights, IL, USA). Nisoldipine and nifedipine‐*d*_6_ were obtained from A2B Chem (San Diego, CA, USA). Nisoldipine‐d_6_ was provided by Chiralix (Nijmegen, the Netherlands). HPLC grade (purity > 98%) solvents dimethyl sulfoxide (DMSO), methanol (MeOH), acetonitrile (ACN), water (H2O), and formic acid (FA) were obtained from Biosolve BV (Valkenswaard, the Netherlands). All chemicals were analyzed without further purification. Target molecules were prepared as a 1 mM solution in DMSO and then diluted to 10 μM solutions in ACN/MeOH/H_2_O and acetic acid with a ratio of 75/12.5/12.4/0.1% (v/v).

### 2.2 Instrumentation Setting and Data Analysis

A Synapt HDMS G2Si (Waters, Milford, MA, USA) equipped with an electrospray ionization source and a travelling wave ion mobility spectrometry (TWIMS) device was used for IMS and MS/MS analysis. The instrument was calibrated using a sodium formate solution. For the calibration of the IMS, a polyalanine standard was used. Sample solutions were injected by direct infusion with a Chemyx fusion 100 syringe pump (Stafford, TX, USA). Positive ion mode was utilized. Detailed instrument parameters are listed in Table S1 in Supporting Information Appendix 1. Mass spectra were acquired within a mass‐to‐charge (m/z) range of 50–800. N_2_ gas was mainly filled in the ion mobility cell but will slightly mix with Helium gas due to the helium cell before the ion mobility cell. Argon gas was used in collision cells for CID‐based fragmentation. Collision energy applied in fragmentation included 5, 10, 15, 20, 25, and 30 eV to make sure precursor ions fragmented completely. Mass spectrometry data were analyzed with MassLynx v4.1. The ATD was analyzed with DriftScope v2.9. Precursor ion scan analysis was performed on Xevo TQ‐S micro Triple Quadrupole Mass Spectrometry (Waters, Milford, MA, USA). Geometry optimization was performed by DFT calculations using ORCA 5.0.1 with B3LYP function and Def‐TZVP basis set. Different proton/sodium locations were tested and the geometries with the lowest energies were reported herein. The transition state was found by NEB‐TS with B3LYP function and Def‐SVP basis set. The found structure was further optimized with Def‐TZVP basis set [31]. The coordinates of optimized structures are provided in Appendix 2 of Supporting Information.

## 3 Results and Discussion

**FIGURE 2:**
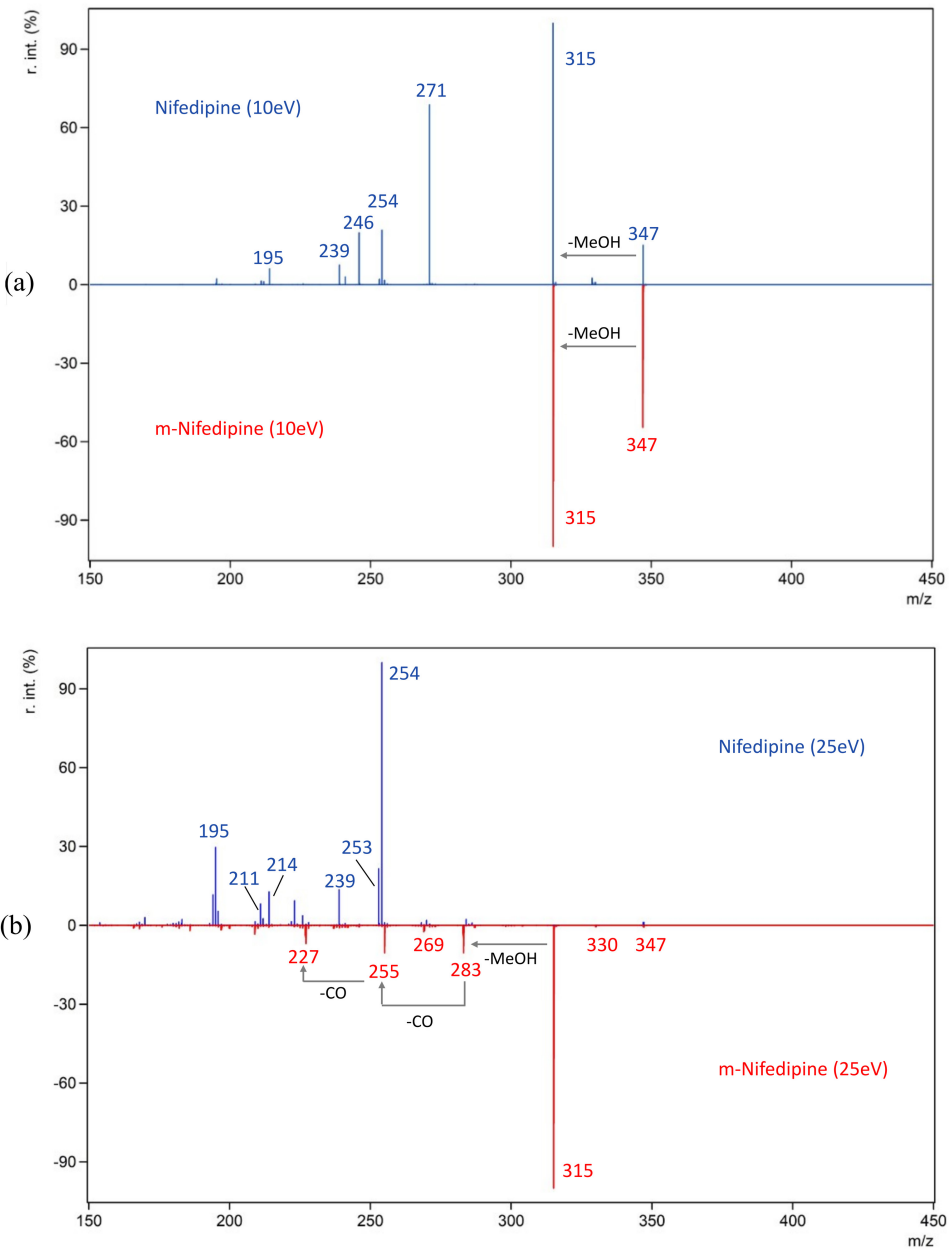
The comparison of fragmentation of protonated nifedipine and *m*‐nifedipine at different collision energies: (a) at 10 eV and (b) at 25 eV.

The MS/MS spectra of nifedipine reported in the literature indicate that the nitro group at the ortho position leads to a significantly different fragmentation mechanism compared with meta positional isomers [24, 25, 32]. Indeed, the MS/MS spectra of nifedipine and *m*‐nifedipine at 10 and 25 eV collision energies are significantly different, as shown in Figure 2. The fragmentation patterns of *m*‐nifedipine and *p*‐nifedipine (Figure S1) are similar, with the major fragments attributed to the typical loss of MeOH and carbon monoxide (CO) from the ester groups.

**FIGURE 3:**
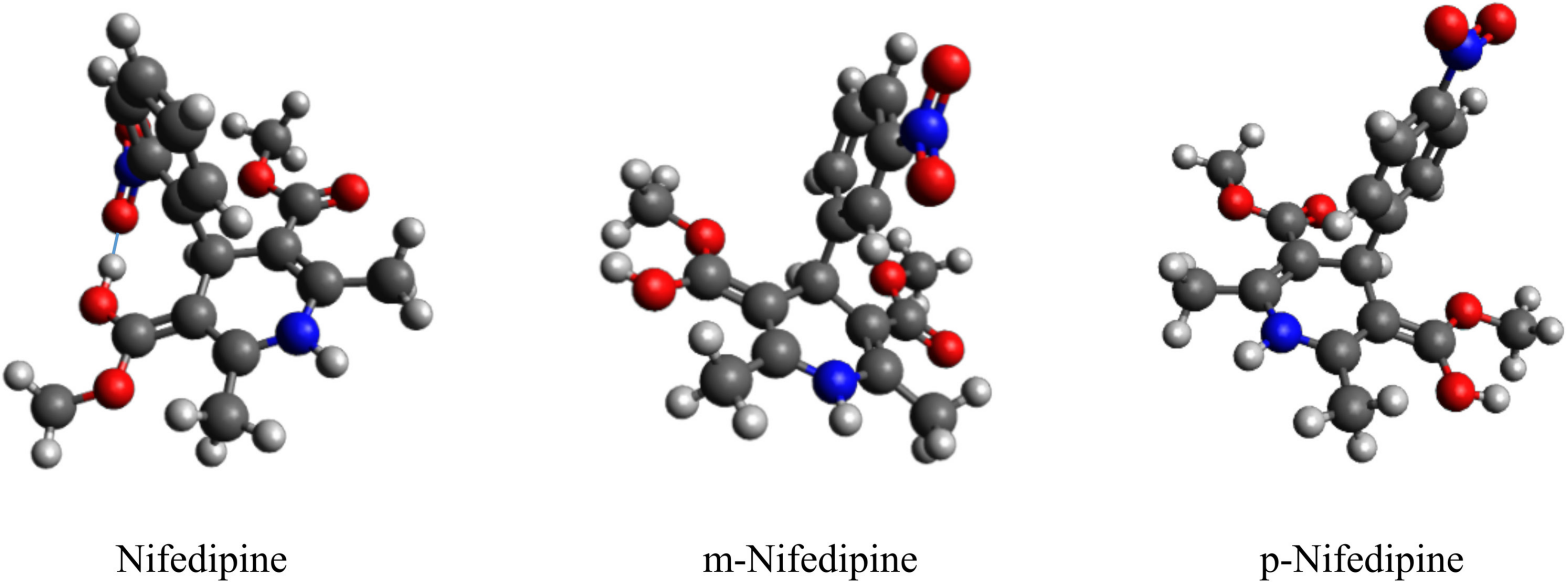
Geometry optimization of nifedipine, *m*‐nifedipine, and *p*‐nifedipine. White: hydrogen; red: oxygen; blue: nitrogen; grey: carbon. The hydrogen bond in nifedipine is labelled as the blue line.

At 10 eV, fragment ions of nifedipine below m/z 315 are more abundant compared with meta‐ and para‐ isomers, suggesting a distinct fragmentation pathway due to the ortho position of the nitro‐group (Figure 2a) [22, 23]. Despite the presence of abundant low‐mass fragments at low collision energy, theoretical calculations reveal that the precursor ion m/z 347 and the fragment ion m/z 315 are more thermodynamically favorable for nifedipine, compared with those ions in meta‐ and para‐ isomers (Table 1). The small spatial distance of the nitro and ester groups in nifedipine appears to facilitate proton stabilization through intramolecular interactions (Figure 3).

**TABLE 1:** Comparison of Gibbs free energy (kcal/mol) of protonated nifedipine, *m*‐nifedipine, *p*‐nifedipine, and their m/z 315 fragments by DFT calculation. The cation with the lowest energies will be treated as zero point and compare with other ions.

	Nifedipine	*m*‐Nifedipine	*p*‐Nifedipine
m/z 347, [**M+**H]^+^	0	+11.59	+12.93
m/z 315	0	+1.48	+3.34

**FIGURE 4:**
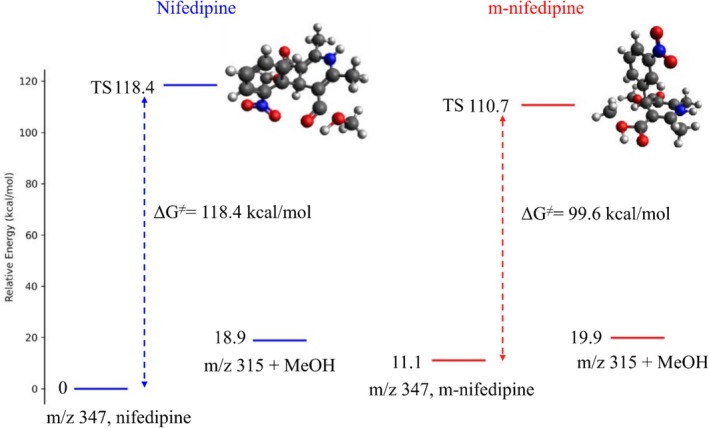
DFT calculation on activation barriers of nifedipine and *m*‐nifedipine.

The calculated activation energies for methanol loss from nifedipine and *m*‐nifedipine show that the energy barrier for nifedipine is 18.8 kcal/mol higher (Figure 4), indicating greater kinetic stability of the nifedipine precursor ion. Although the m/z 315 fragment ion of nifedipine is only marginally more thermodynamically stable than that of *m*‐nifedipine (by 1.48 kcal/mol, Table 1), this small energy difference alone does not account for the pronounced differences observed in Figure 2. Instead, the results suggested that the observed fragmentation patterns are primarily governed by the kinetic stability of m/z 315 in nifedipine and its isomers.

**FIGURE 5:**
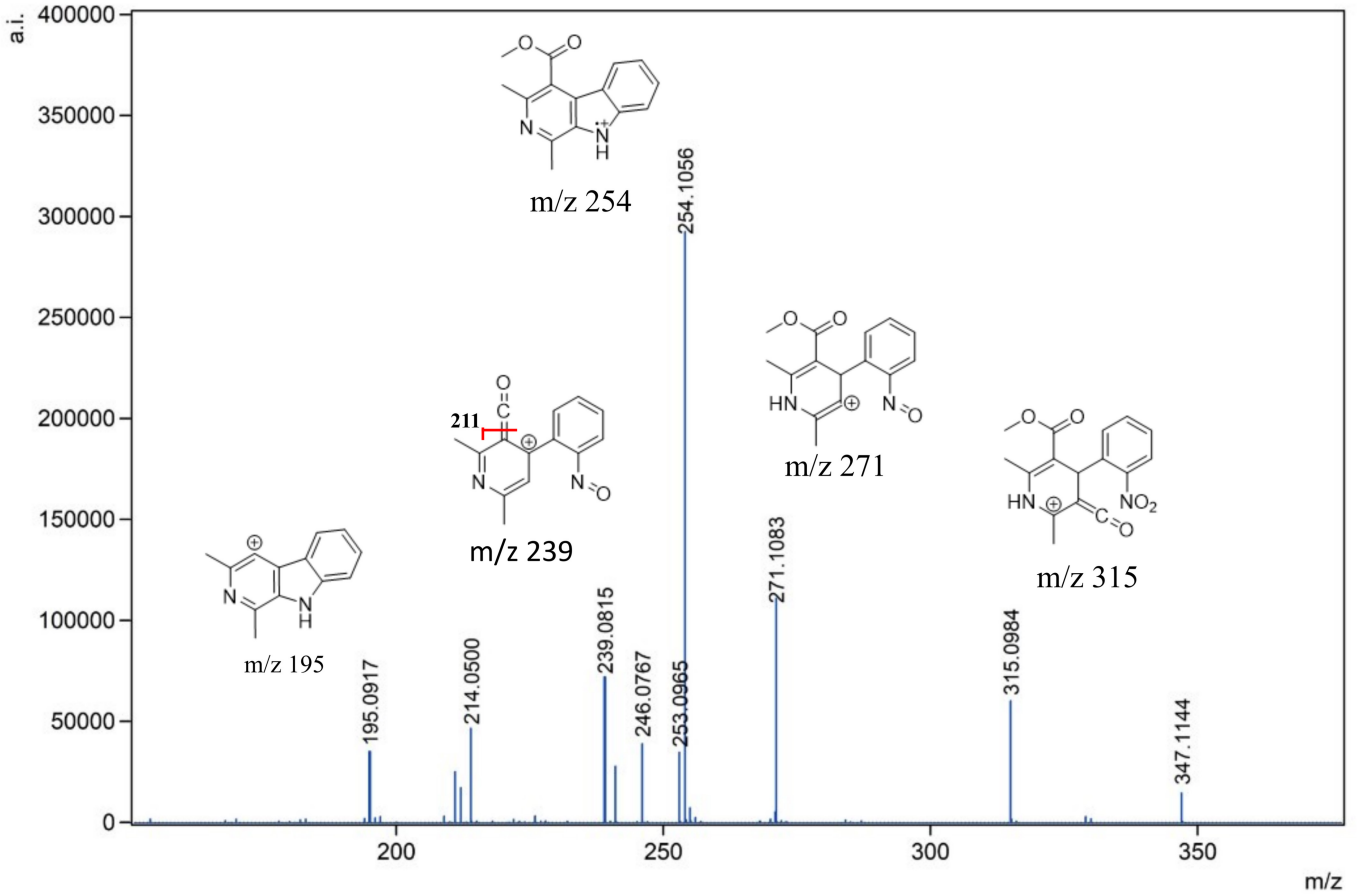
MS/MS spectrum of [M+H]^+^ at collision energy of 15 eV with proposed structures in literature [20].

The MS/MS spectrum of nifedipine at 15 eV shows prominent fragment ions at m/z 315, m/z 271, m/z 254, m/z 239, m/z 211, and m/z 195 (Figure 5). The ion at m/z 271 is supposed to originate from m/z 315 due to the rearrangement and the loss of CO_2_. The fragment ion at m/z 254 is subsequently formed through the loss of the OH• radical, a process enabled by the nitro group at the ortho position [20]. Additional fragment ions at m/z 253, m/z 246, m/z 241, and m/z 214 have not been extensively discussed in previous studies and will be discussed in the following sections. Lastly, the minor fragment at m/z 330 is a well‐known radical loss of OH• from the protonated molecule.

To further understand the contribution of the ortho‐nitro group on the unique fragmentation process, theoretical calculations of key fragmentation steps, particularly the transition from m/z 315 to m/z 271, are crucial for annotating the structures and assessing the relationships between the fragment ions. This is especially important, as the unambiguous proof of the fragmentation pathways and ion structures is still lacking, and the relationships between the fragment ions require further confirmation [20]. In this research, the fragmentation pathways of the protonated molecule ([M+H]^+^) and the sodium adduct ions ([M+Na]^+^) of nifedipine are further investigated, emphasizing the influence of the ortho effect on the rearrangement and the associated radical loss.

### 3.1 Assessment of the Fragmentation Pathways

**FIGURE 6:**
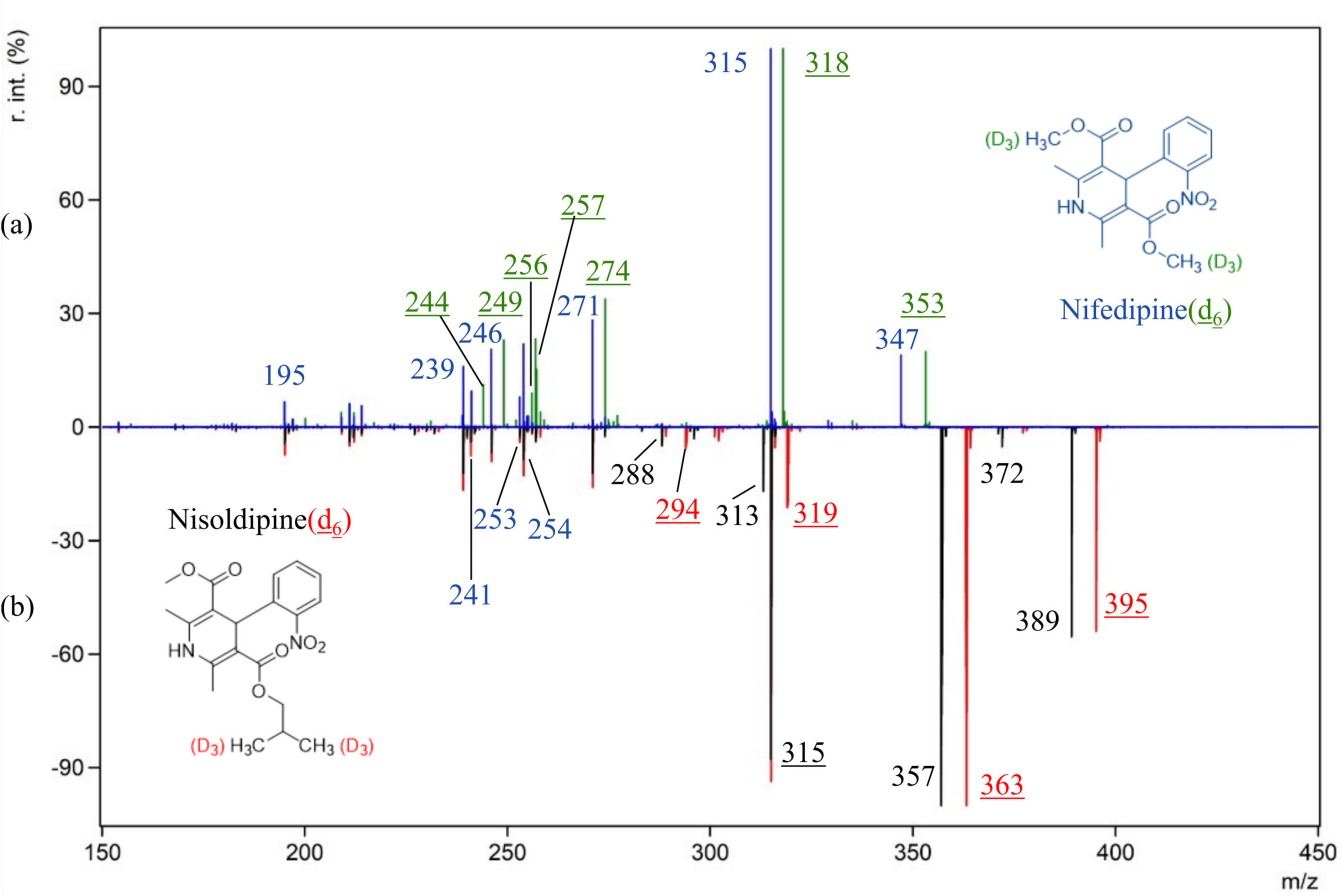
the comparisons of MS/MS spectrum of protonated nifedipine analogues at 5 eV: (a) nifedipine (blue) and nifedipine‐*d*_6_ (green with underline) and (b) nisoldipine (black) and nisoldipine‐*d*_6_ (red with underline).

To systematically study the ortho effect, nifedipine analogues like aranidipine and nisoldipine, as well as deuterium‐labelled compounds, were analyzed. The asymmetric ester chains and deuterium atoms lead to the mass shifts of fragment ions. They provide additional evidence for the fragmentation pathway of nifedipine. The MS/MS spectra of nifedipine‐*d*_6_, nisoldipine, and nisoldipine‐d_6_ at 5 eV are depicted and compared in Figure 6. The MS/MS spectrum of nisoldipine and aranidipine at higher collision energy (10 eV) is shown in Figure S2 to indicate the mass shifts of peaks m/z 254, m/z 253, m/z 246, and m/z 241. The detailed list of the fragment ions and their assignment can be found in Table S2.

As expected, next to nifedipine, nisoldipine and aranidipine have similar fragmentation patterns due to the ortho effect. The fragment ions below m/z 239 are comparable and the characteristic fragment ions at m/z 357 and m/z 315, resulting from the loss of MeOH or C_4_H_9_OH, mimic the loss of either of the two MeOH moieties from nifedipine. The ion m/z 313 in nisoldipine corresponds to m/z 271 in nifedipine and the m/z 288 has a similar structure to m/z 246 due to the presence of a different side chain. Both ions exhibit a mass shift of 6 Da in nisoldipine‐*d*_6_, appearing at m/z 319 and m/z 294. The formation of a potential fragment ion at m/z 283, which would result from the subsequent loss of a second methanol moiety, is not observed.

For aranidipine, compared with nisoldipine, the ratio between the loss of longer side chain (m/z 315) and the loss of MeOH (m/z 357) is more predominant at 10 eV (Figure S2). A possible explanation is the relatively lower heat of formation of CH_3_COCH_2_OH compared to iso‐butyl alcohol. Moreover, in addition to nisoldipine and nifedipine, the loss of the OH• radical, leading to the m/z 372 fragment ion, is significantly enhanced in aranidipine, which may be attributed to the ketone group in the side chain.

Furthermore, fragment ions at m/z 296, m/z 295, and m/z 283 are also detected at low intensity in the MS/MS spectra of the analogues, corresponding to m/z 254, m/z 253, and m/z 241, respectively. Overall, the mass shifts detected in ortho‐analogues and deuterium‐labelled compounds are consistent with the proposed structures shown in Figure 5.

### 3.2 Analysis of Fragment's Relationship

**FIGURE 7:**
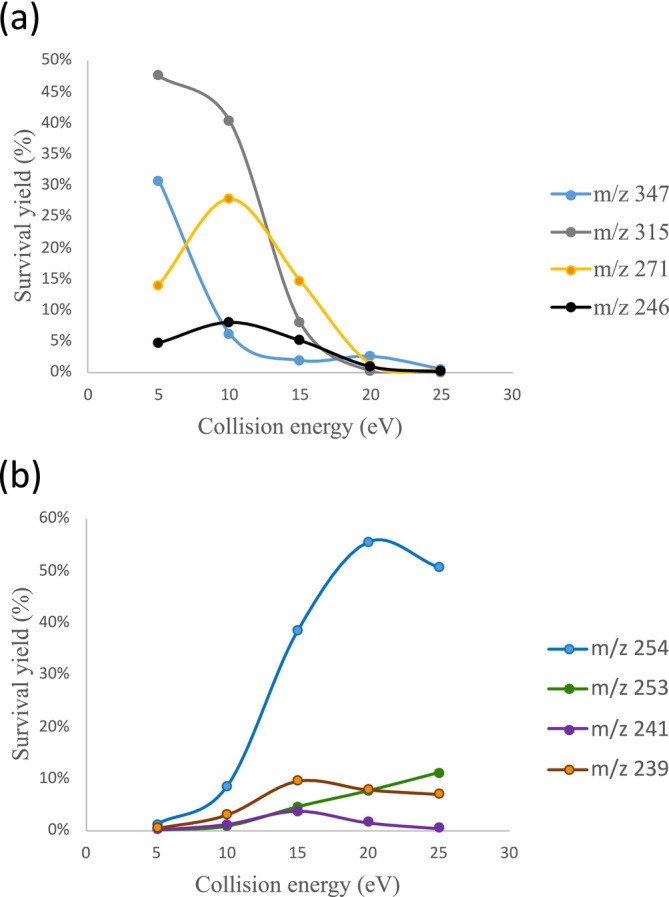
The survival yield of fragments at different collision energies. (a) Ion m/z 347, m/z 315, m/z 271, and m/z 246 and (b) ion m/z 254, m/z 253, m/z 241, and m/z 239.

The abundance of fragment ions is linked to their stability (i.e., degree of charge delocalization) and their survival yield of the ions [33, 34]. In Figure 7a, the survival yields are calculated according to the Equation (1) and plotted as a function of collision energies, where *I*_fi_ is the intensity of the interested fragment, *I*_p_ is the intensity of the remaining precursor ion, and Σ*I*_f_ is the sum of the intensities of all fragments [34]. The behaviour of the precursor ion (m/z 347) and the fragment ion m/z 315 are shown, revealing their rapid decline between 5–10 eV and 10–15 eV, respectively. Likewise, the fragment ions at m/z 271 and m/z 246 act as intermediates and undergo further fragmentation at collision energies above 10 eV. In Figure 7b, the intensity changes of the fragment ions at m/z 254, m/z 253, m/z 241, and m/z 239 are presented, originating from m/z 315 and m/z 271 ions. The fragments at m/z 241 and m/z 239 show similar trends and are likely formed from m/z 271, although they are less favorable pathways, involving the loss of nitric oxide or methanol, respectively. The fragment m/z 253 is expected to have a structure similar to that of m/z 254 but is formed by losing H_2_O from m/z 271.

(1) $$\text{Survival Yield}\;\left(\%\right)=\frac{I_{\mathrm{fi}}}{I_{p}+\Sigma I_{f}}$$

**FIGURE 8:**
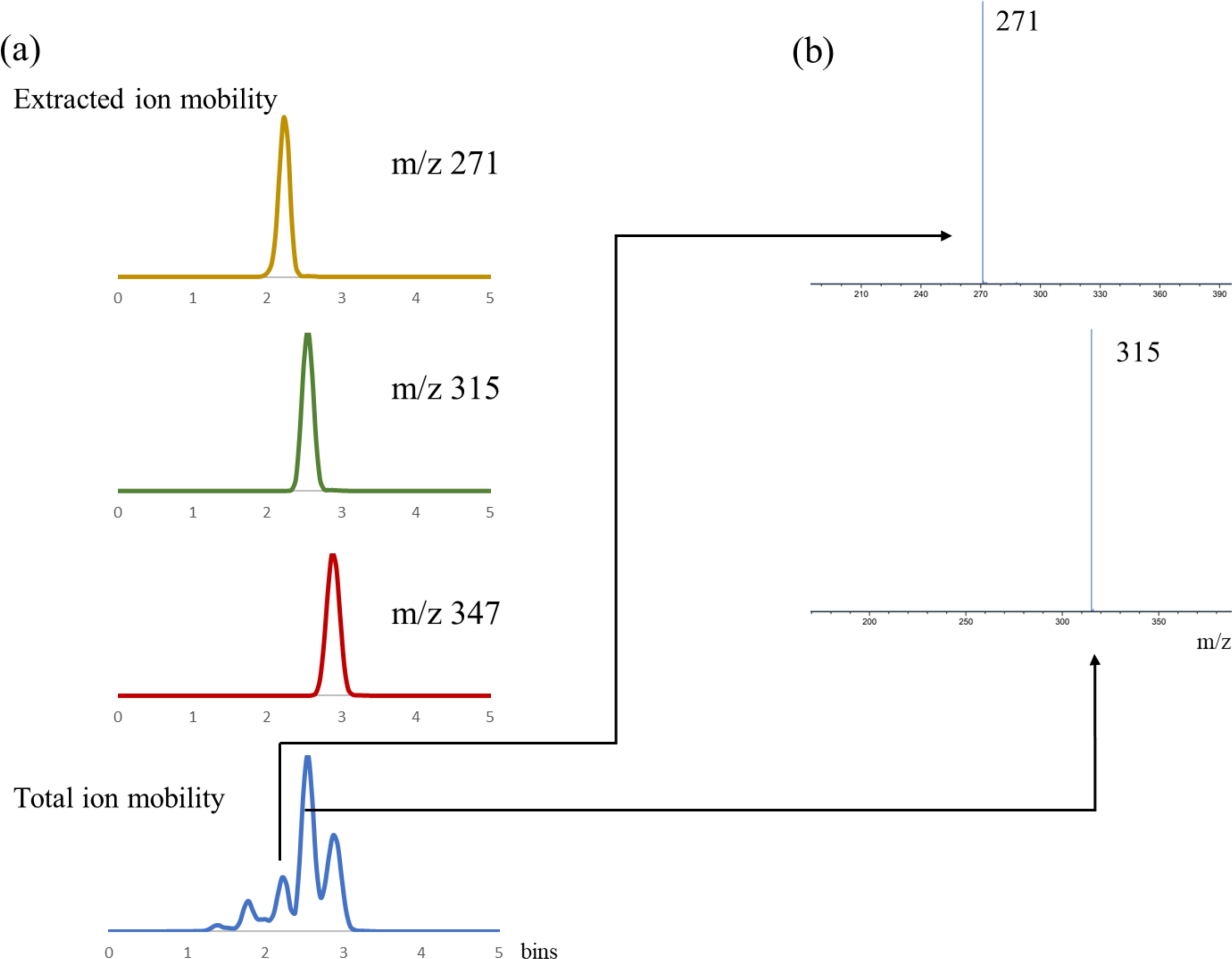
CID‐IMS analysis of nifedipine. (a) The comparison of total ion mobility of nifedipine fragmentation and extracted ion mobility of precursor ion m/z 347 and fragments m/z 315 and m/z 271. (b) The mass information in specific ion pack of total ion mobility.

In the Waters Synapt G2Si instrument, the ion mobility cell is positioned between two collision cells, known as the trap cell and the transfer cell. This configuration allows the IMS separation of fragment ions formed prior to the IMS cell, as well as MS/MS analysis of ions exiting the IMS cell. If fragmentation occurs prior to the IMS (CID‐IMS), that is, within the trap cell, both precursor and fragment ions are separated by the travelling wave based on differences in their mobility and/or ATD. As an example, the fragments of nifedipine are separated by IMS, each exhibiting a distinct ATD, as shown in Figure 8a. Because all fragments are separated by IMS, the mass information within ion packet of the total ion mobility spectrum mainly corresponds to individual fragment ions (Figure 8b).

**FIGURE 9:**
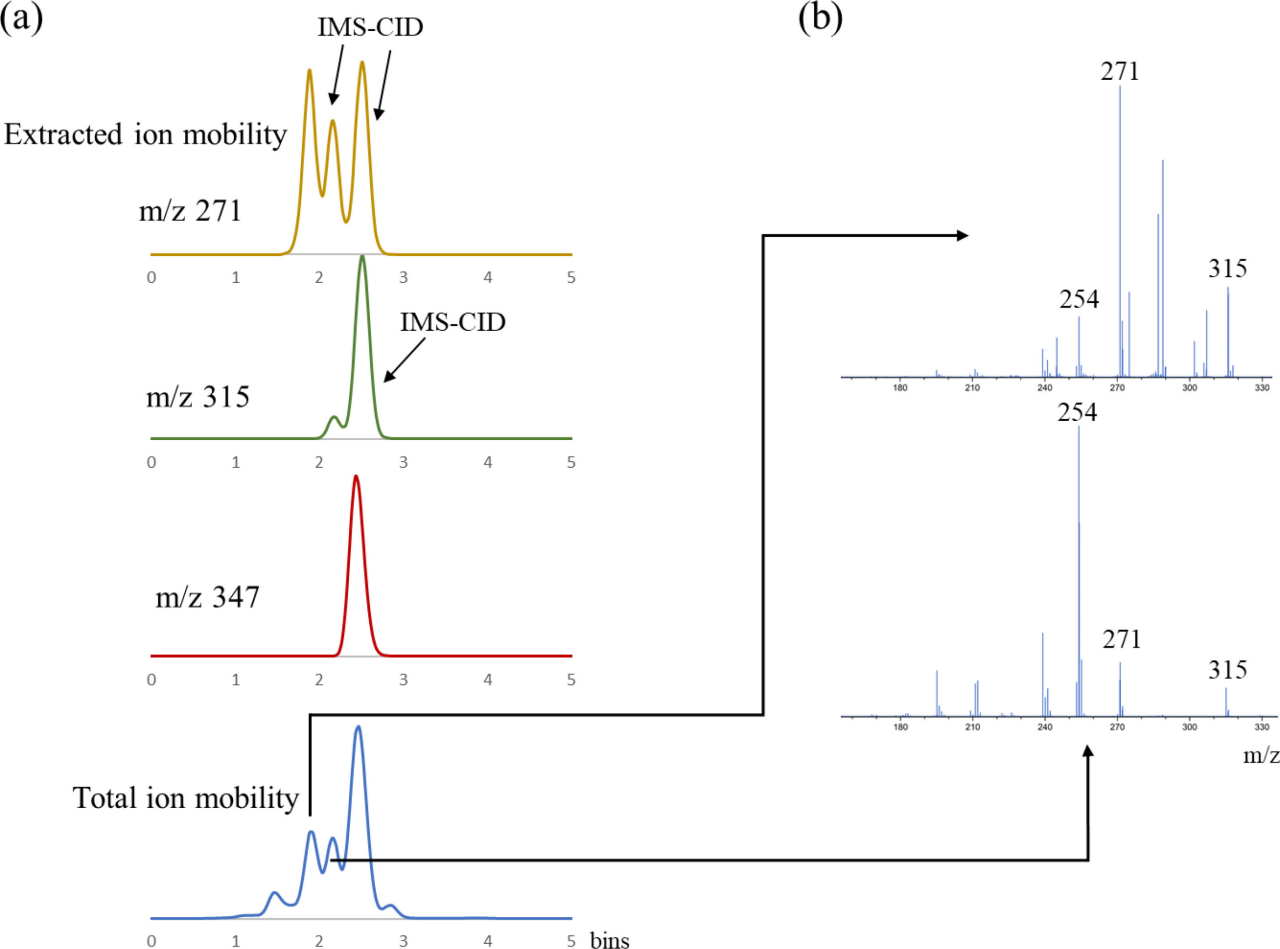
IMS‐CID analysis of nifedipine. (a) The comparison of total ion mobility of nifedipine fragmentation and extracted ion mobility of precursor ion m/z 347 and fragments m/z 315 and m/z 271. (b) The mass information in specific ion pack of total ion mobility.

Instead, if fragmentation is activated after ion mobility separation (IMS‐CID), that is, in the transfer cell, the resulting fragment ions remain the same ATD values as their precursors. Figure 9a demonstrates that the ion mobility spectrum of m/z 271 contains peaks at the same arrival time as m/z 315 and m/z 347. This indicates that m/z 315 originates from the protonated molecule at m/z 347 and that m/z 271 is generated from m/z 315. The significance of this approach has been reported in a previous study, where IMS‐MS/MS analysis was used to assign two isomeric products of a hetero Diels‐Alder reaction. Combined with theoretical calculations, this strategy enabled the annotation of the ion mobility peak [35]. It should be noted that m/z 271 and m/z 315 still exhibit their own distinct peaks generated via CID‐IMS, as shown in Figure 8a. Although no additional collision energy is applied prior to IMS, fragmentation may occur because of the collisions or electrical potential during ion transfer before or within the IMS cell. Unlike controlled CID‐IMS experiments, this type of fragmentation does not occur at a defined time point, meaning that ion mobility peaks may overlap with those of other ions. As illustrated in Figure 9b, the ion packets of m/z 315 and m/z 271 contain not only their respective ions but also contributions from other ions, even including ions with higher m/z values. Nevertheless, combining IMS‐CID with CID‐IMS allows improved interpretation of fragment relationships.

**FIGURE 10:**
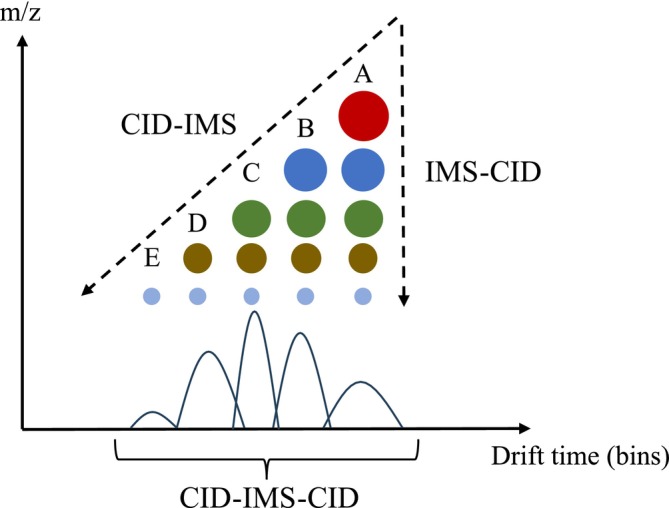
The illustration of CID‐IMS‐CID. The sizes of the ions are corresponding to the mass of the ion.

Figure 10 illustrates the CID‐IMS‐CID workflow. The resulting ion mobility peak excludes ions with m/z values higher than the fragment of interest while retaining its subsequent fragments. This strategy is applied here to unravel the fragment relationships of m/z 271. The precursor ion at m/z 347 is first selected in the quadrupole and fragmented in first collision cell at 5 eV to enhance the intensity of m/z 271. The resulting ion mixture is then separated by IMS and further fragmented with higher collision energy (15 eV) in the second collision cell (CID‐IMS‐CID).

**FIGURE 11:**
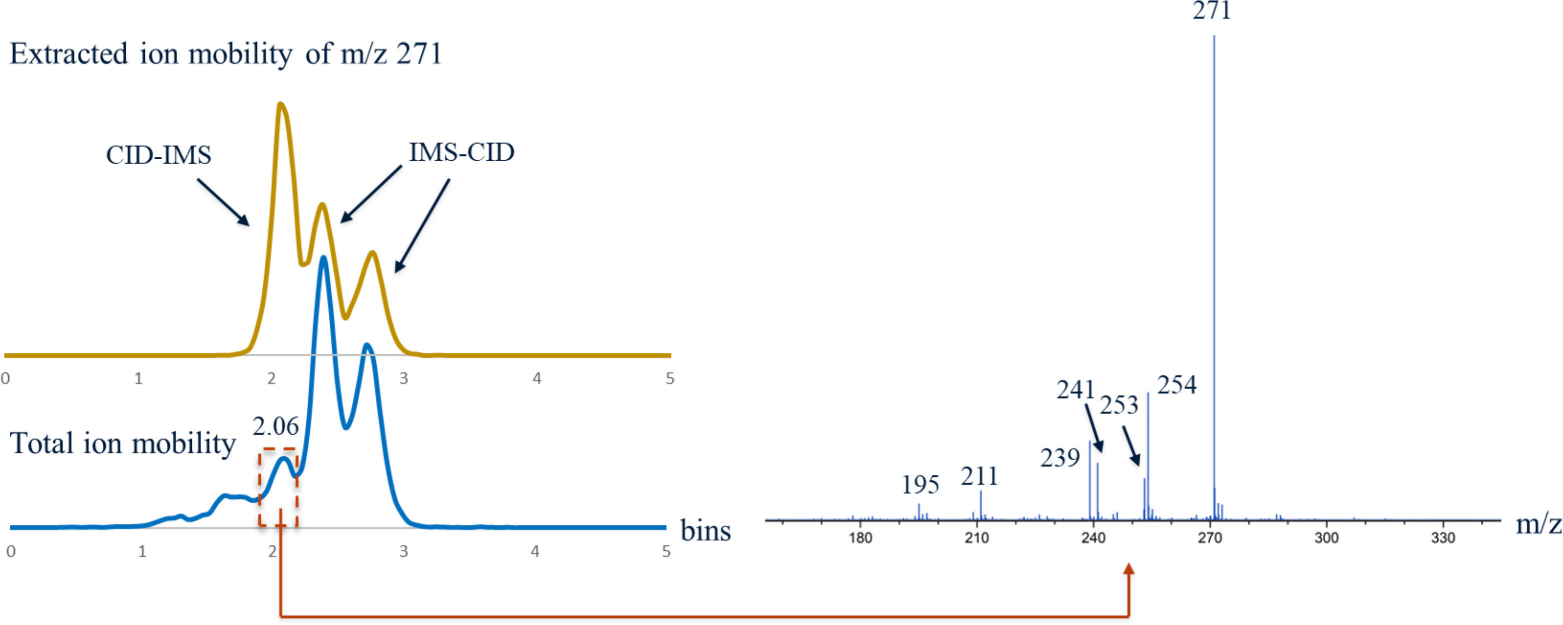
CID‐IMS‐CID analysis of m/z 347. The ion mobility of m/z 271 is extracted from total ion mobility and the mass information of peak at 2.06 bins of total ion mobility is analyzed.

In Figure 11, the ion mobility of the m/z 271 fragment ions is illustrated. As discussed previously, the ion mobility peak observed in IMS‐CID experiments identifies m/z 271 as a fragment originating from m/z 315. The ion mobility peak at 2.06 bins represents a unique ion packet corresponding to m/z 271 generated by CID‐IMS. By extracting and overlaying the ion mobility data from the total ion mobility spectrum, the distribution of the ion packet at 2.06 bins for m/z 271 can be assigned. Moreover, the mass spectral information associated with the mobility peak at 2.06 bins reveals that fragment ions at m/z 254, m/z 253, m/z 241, m/z 239, m/z 211, and m/z 195 originate from m/z 271. The fragment ion at m/z 246 is not observed in this spectrum. Further CID‐IMS‐CID analysis of m/z 246 indicates that it is directly generated from m/z 347, while m/z 214 is the fragment of m/z 246 by losing MeOH (Figure S3). This assignment is supported by deuterium isotope labelling in Figure 6. However, the proposed structure and detailed fragmentation mechanism remain unclear. Additional data of PIS proving the relationships are provided in Figure S4.

### 3.3 The Fragmentation Mechanism of Nifedipine

**SCHEME 1:**
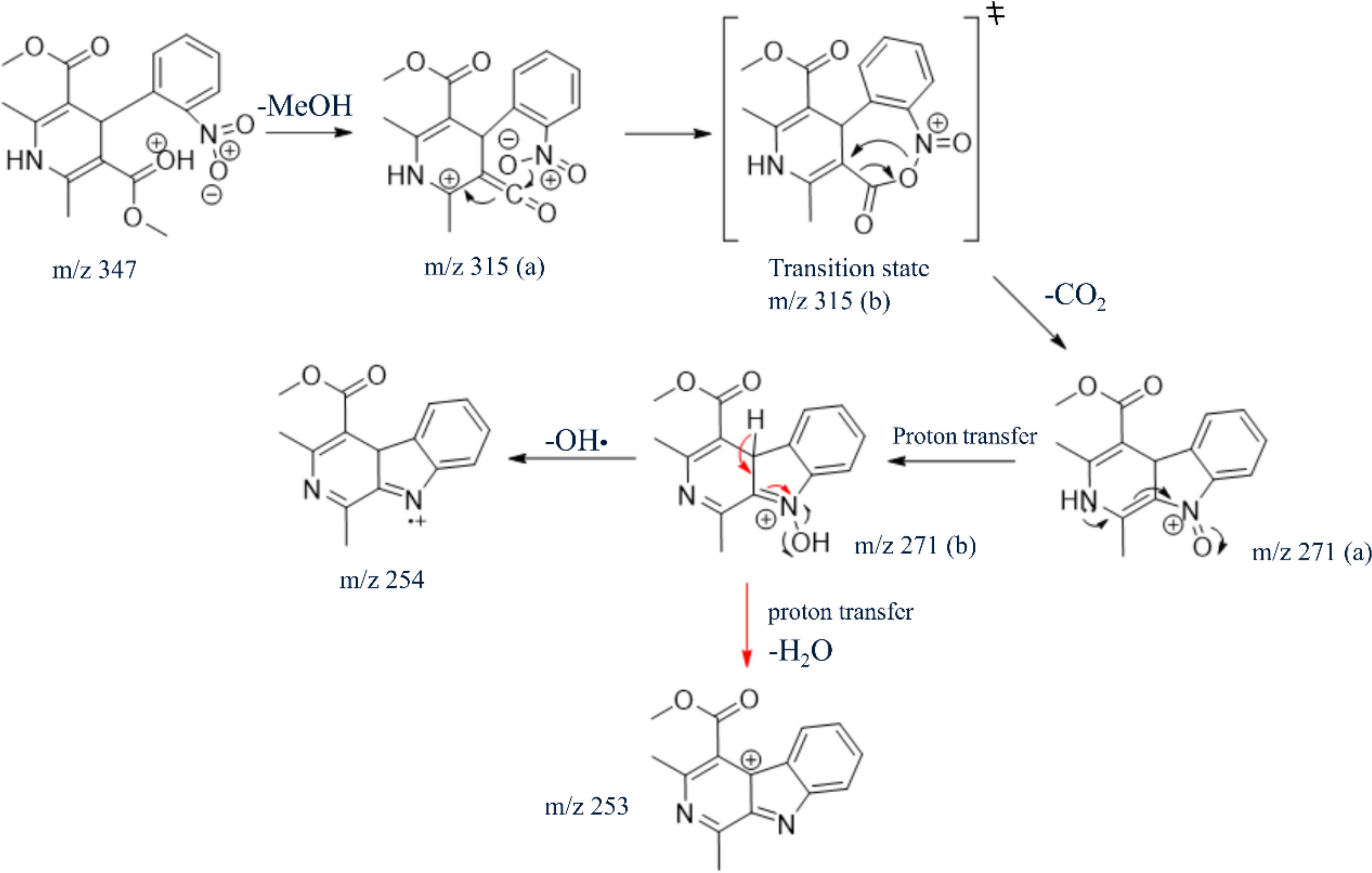
proposed fragmentation pathway of protonated nifedipine.

Based on the reported structures and the data presented above, Scheme 1 provides a more comprehensive and systematic overview of the fragmentation pathways, incorporating the ortho effect and supported by theoretical calculations. A revised structure for m/z 271 is proposed after geometry optimization, differing from the structure previously reported in the literature. The formation of a seven‐membered ring is essential for the generation of m/z 271 via CO_2_ loss. The ATDs of m/z 315 and m/z 271 are similar to that of the precursor ion m/z 347, suggesting well‐defined conformations for these fragment ions.

**FIGURE 12:**
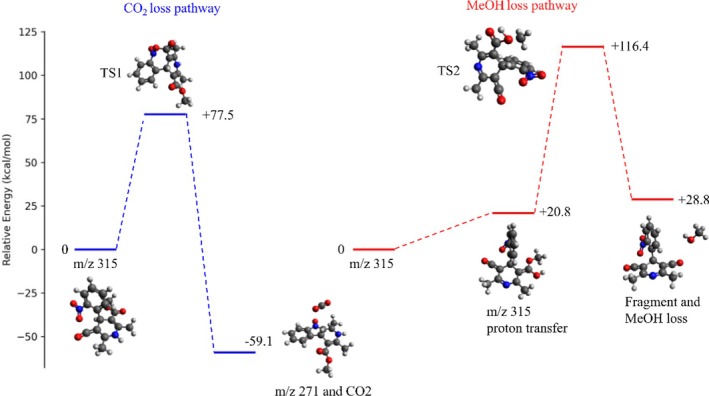
Comparison of nifedipine fragmentation processes for observed CO_2_‐loss pathway (m/z 315 to m/z 271) and imagined MeOH‐loss pathway (m/z 315 to m/z 283).

Considering the relative high intensity and corresponding stability of m/z 315 and m/z 271, the proposed structures for m/z 315 (a) and m/z 271 (a) are considered plausible. In contrast, m/z 315 (b) is unstable and directly converts to m/z 271 (a) during geometry optimization, indicating that it represents a transition state rather than a stable intermediate. DFT modeling of the fragmentation pathway from m/z 315 (a) and m/z 271 (a), using m/z 315 (b) as the transition state, results in the mechanism shown in Figure 12. For comparison, direct loss of MeOH from the protonated molecule without rearrangement was also calculated. The activation barrier for CO_2_ loss is 38.9 kcal/mol lower than that for MeOH loss, indicating higher stability of the m/z 315 fragment in *m*‐nifedipine and explaining the differences in fragmentation patterns shown in Figure 2. Moreover, the generation of m/z 254 involved an even‐to‐odd fragmentation, resulting in a radical cation through the loss of OH• radical from m/z 271. As discussed before, the formation of m/z 254 is highly favored compared to competing pathways leading to m/z 253 fragment (loss of H_2_O), m/z 241 (loss of NO), and m/z 239 (loss of MeOH).

### 3.4 Ortho Effect on Sodium Adducts of Nifedipine

**FIGURE 13:**
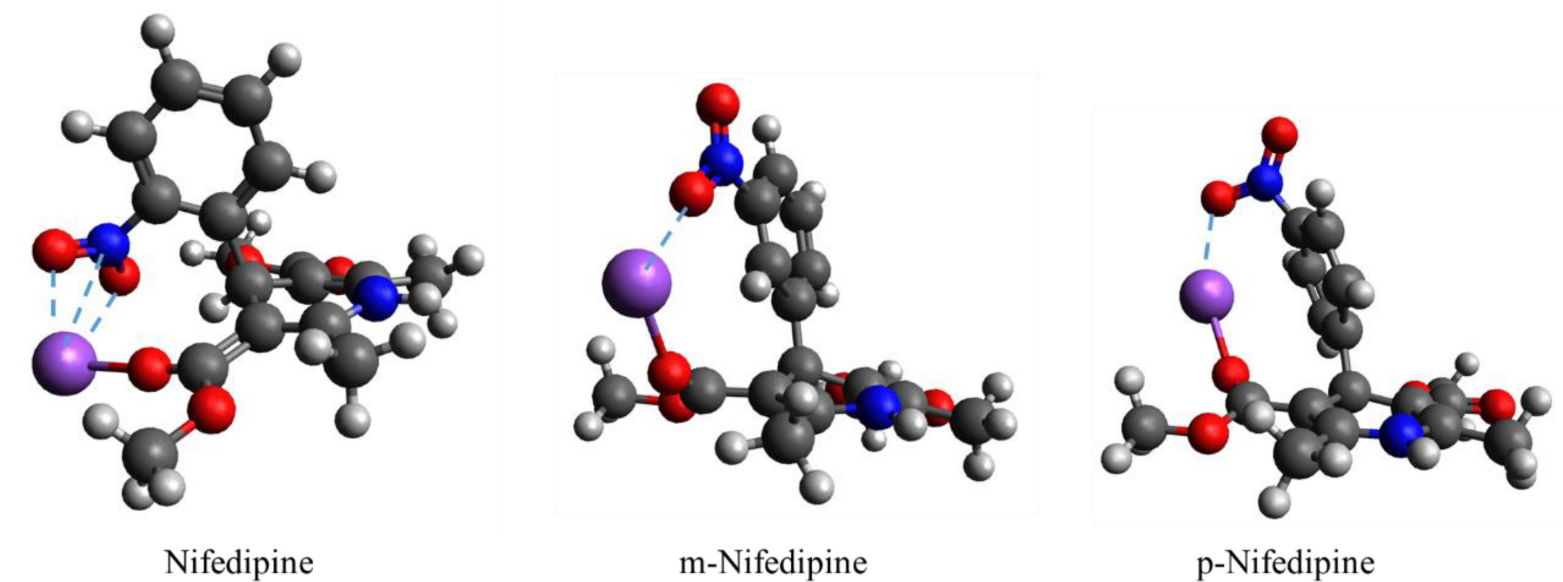
geometry optimization of sodium adducts of nifedipine, *m*‐nifedipine, and *p*‐nifedipine. The intramolecular interactions between sodium atom and NO_2_ group are labelled as dash line.

**FIGURE 14:**
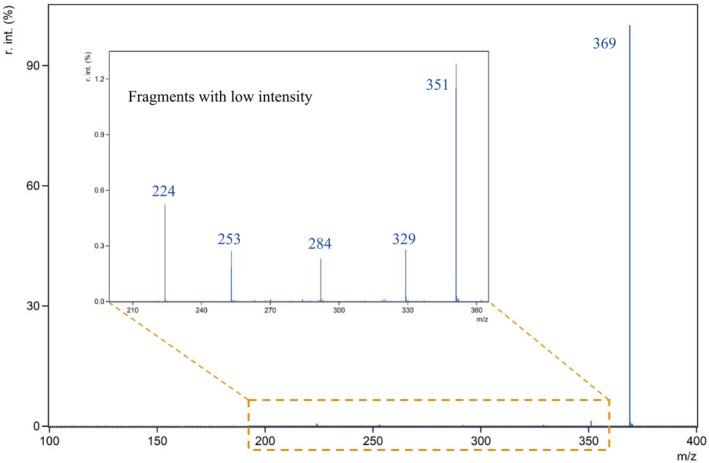
Fragmentation at 15 eV of nifedipine in sodium adduct form.

In addition to the protonated molecule of nifedipine, the fragmentation behavior of [M+Na]^+^ was also investigated, as it was hypothesized to be influenced by the ortho effect. The meta‐ and para‐position isomers do not show significant fragmentation at low energy. Instead, the precursor ion intensity decreases significantly when the collision energy increases from 15 to 20 eV, without the formation of notable fragment ions. This suggests that, at higher collision energies, the sodium ion is stripped away, leaving a neutral molecule. Interestingly, the [M+Na]^+^ ion of nifedipine still remains as the most thermodynamically stable cation among the three isomers, likely due to increased interactions with the NO_2_ group (Figure 13). Nonetheless, compared to the protonated molecule, it generates a number of interesting fragment ions at 15 eV (Figure 14).

**SCHEME 2:**
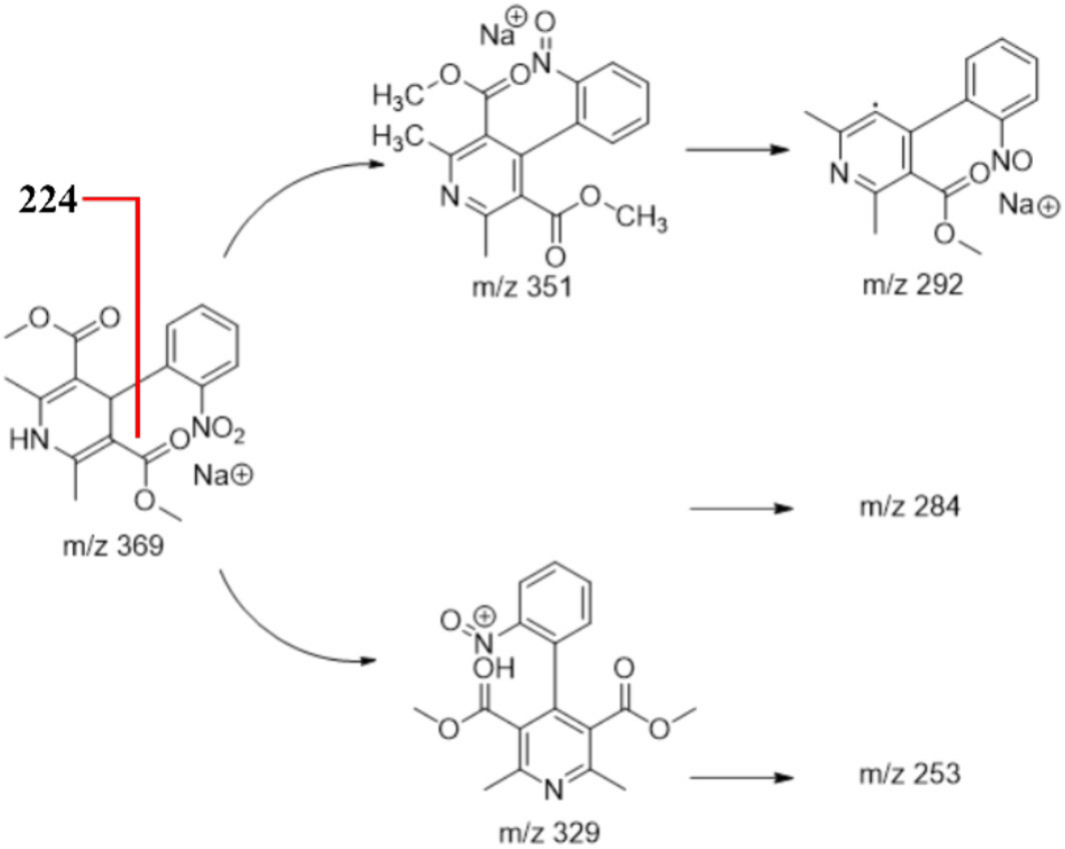
Proposed fragmentation pathway of nifedipine sodium adduct, [M+Na]^+^.

Analysis of aranidipine, nisoldipine, and nifedipine‐*d*_6_ further confirms clear mass shifts in these fragment ions (Figure S5). The formation of m/z 351 is likely due to the loss of water after proton rearrangement from the nitrogen and carbon atoms toward the nitro group (Scheme 2). Because all labile protons are removed during the generation of m/z 351, this fragment can lose COOCH_3_• radical to form m/z 292, most probably by homolytic cleavage. Ion mobility analysis of m/z 329 and m/z 224 suggests that both ions are directly generated from m/z 369 (Figure S6). The formation of m/z 224 is likely due to the cleavage of the bond between the two rings, with the charge retained on the dihydropyridine moiety while sodium is lost with nitrobenzene. The fragment ion m/z 329 is formed via loss of sodium hydroxide.

Although ion mobility signals are relatively weak, the data suggest that m/z 329 can further generate m/z 284 and m/z 253 (Figure S6). Besides, DFT calculations also indicate that sodium adducts formed after proton transfer are more stable (Figure S7), explaining the observed loss of H_2_O or NaOH. Nevertheless, the fragmentation pathways proposed in Scheme 2 are more speculative than those in Scheme 1 and require further investigation. Preliminary gas‐phase IR spectroscopy experiments suggest the structures of fragment ion at m/z 253 differ between [M+H]^+^ and [M+Na]^+^ species. Future studies are expected to provide further insight into the precise structures of fragment ions.

## 4 Conclusions

The fragment ions of nifedipine reported in the literature are consistent with the data obtained in this study, including results from analogues, stable isotope‐labelled molecules, ion mobility, PIS experiments and DFT calculations. In addition, fragment structures originating from [M+H]^+^ and [M+Na]^+^ that have not been previously discussed in the literature were investigated, providing further evidence for the proposed fragmentation pathways. Furthermore, DFT calculations support the bond rearrangement involved in the fragmentation mechanism leading to m/z 271 ion. Radical cations are observed in both the [M+H]^+^ and [M+Na]^+^ of nifedipine. The role of the NO_2_ group at the ortho position, that is, the so‐called ortho effect, in facilitating radical loss is further reinforced. Additional evidence supporting the influence of the ortho effect on the fragmentation of sodium adduct ions is also presented. Consequently, both *m*‐ and *p*‐nifedipine predominantly undergo Na^+^ stripping rather than forming characteristic fragment ions.

A key aspect of this study is the combined application of structurally well‐defined analogues, deuterium isotope labeling and multiple experimental fragmentation strategies, including CID‐IMS and IMS‐CID. The overall ion mobility diagram, which contains information comparable to PIS in triple quadrupole mass spectrometry, has demonstrated its added value in molecular structure analysis. Therefore, analysis of the ATD of each fragment ion is crucial for elucidating fragment structures and assessing the possible existence of various resonance or isomeric structures. Although this capability has not yet been fully explored, it represents an important direction for future research.

## Funding

This work was supported by the Nederlandse Organisatie voor Wetenschappelijk Onderzoek (18433).

## Supporting information




**Table S1:** Parameters of Waters Synapt G2Si HDMS system for sample analysis.
**Figure S1:** Fragmentation of protonated p‐nifedipine with proposed ion structures. p‐nifedipine has similar fragmentation as m‐nifedipine but fragment due to the loss of OH• has higher intensity.
**Figure S2:** Fragmentation of protonated Nisoldipine (a) and Aranidipine (b) at 10 eV collision energy with proposed structures of fragments.
**Table S2:** Lists of observed fragments for protonated nifedipine (d6), nisoldipine (d6), and aranidipine.
**Figure S3:** CID‐IMS‐CID analysis of m/z 246.
**Figure S4:** Precursor ion scan of fragments of protonated nifedipine: (a) m/z 271; (b) m/z 254; (c) m/z 253; (d) m/z 241; (e) m/z 239; (f) m/z 211.
**Figure S5:** MS/MS fragmentation of sodium adduct of (a) nifedipine‐d6, (b) aranidipine, (c) nisoldipine, and (d) nisoldipine‐d6 at 15 eV collision energy with proposed structures of fragments.
**Figure S6:** Extracted ion mobility of sodium adduct fragments of nifedipine in analysis of CID‐IMS‐CID.
**Figure S7:** Sodium adduct of nifedipine after proton transfers, where grey atom is carbon, red atom is oxygen, white atom is hydrogen, blue atom is nitrogen and purple atom is sodium. Sodium cation is stabilized by oxygen atoms (dash line).

## Data Availability

The data that support the findings of this study are available from the corresponding author upon reasonable request.
