# Supplementary material for: A Combined MS/MS and IMS Study Into the Fragmentation Pathway of Nifedipine
Source: J Mass Spectrom. 2026 Jun 11;61(7):e70071. doi: 10.1002/jms.70071 (PMC13254695; doi:10.1002/jms.70071)
Supplement: Supplementary file 1 — Table S1: Parameters of Waters Synapt G2Si HDMS system for sample analysis. Figure S1: Fragmentation of protonated p‐nifedipine with proposed ion structures. p‐nifedipine has similar fragmentation as m‐nifedipine but fragment due to the loss of OH• has higher intensity. Figure S2: Fragmentation of protonated Nisoldipine (a) and Aranidipine (b) at 10 eV collision energy with proposed structures of fragments. Table S2: Lists of observed fragments for protonated nifedipine (d6), nisoldipine (d6), and aranidipine. Figure S3: CID‐IMS‐CID analysis of m/z 246. Figure S4: Precursor ion scan of fragments of protonated nifedipine: (a) m/z 271; (b) m/z 254; (c) m/z 253; (d) m/z 241; (e) m/z 239; (f) m/z 211. Figure S5: MS/MS fragmentation of sodium adduct of (a) nifedipine‐d6, (b) aranidipine, (c) nisoldipine, and (d) nisoldipine‐d6 at 15 eV collision energy with proposed structures of fragments. Figure S6: Extracted ion mobility of sodium adduct fragments of nifedipine in analysis of CID‐IMS‐CID. Figure S7: Sodium adduct of nifedipine after proton transfers, where grey atom is carbon, red atom is oxygen, white atom is hydrogen, blue atom is nitrogen and purple atom is sodium. Sodium cation is stabilized by oxygen atoms (dash line). [file JMS-61-e70071-s001.docx]

Supporting information: **A COMBINED MS/MS AND IMS STUDY INTO THE FRAGMENTATION PATHWAY OF NIFEDIPINE**

Peiliang Han ^1^, Newton Thomassen ^2^, Maarten Honing ^1^

^1^ Maastricht Multimodal Molecular imaging (M4i) Institute, Division of Imaging Mass Spectrometry Maastricht University, Universiteitssingel 50, 6229 ER, Maastricht, The Netherlands.

^2^ Aachen Maastricht Institute Biobased Materials (AMIBM), Brightlands Chemelot Campus, Urmonderbaan 22, 6167 RD, Geleen, The Netherlands.

*To whom correspondence should be addressed:

m.honing@maastrichtuniversity.nl Phone: +31629368323

Appendix 1: experimental data and spectra

1. Table S1:parameters of Waters Synapt G2Si HDMS system for sample analysis

| **Parameters** | **Positive mode** |
| --- | --- |
| Capillary voltage (kV) | 3.0 |
| Sampling cone voltage (v) | 25 |
| Source offset | 55 |
| Source temperature (*°c*) | 100 |
| Desolvation gas temperature (*°*C) | 250 |
| Desolvation gas flow (L/Hr) | 600 |
| Nebulizer gas pressure (bar) | 5.3 |
| Trap gas flow (ml/min) | 2 |
| LM resolution | 16.0 |
| Wave velocity (m/s) | 550 |
| Wave height (v) | 24 |
| IMS gas rate (ml/min) | 60 |

1. Figure S1: Fragmentation of protonated p-nifedipine with proposed ion structures. p-nifedipine has similar fragmentation as m-nifedipine but fragment due to the loss of OH• has higher intensity.


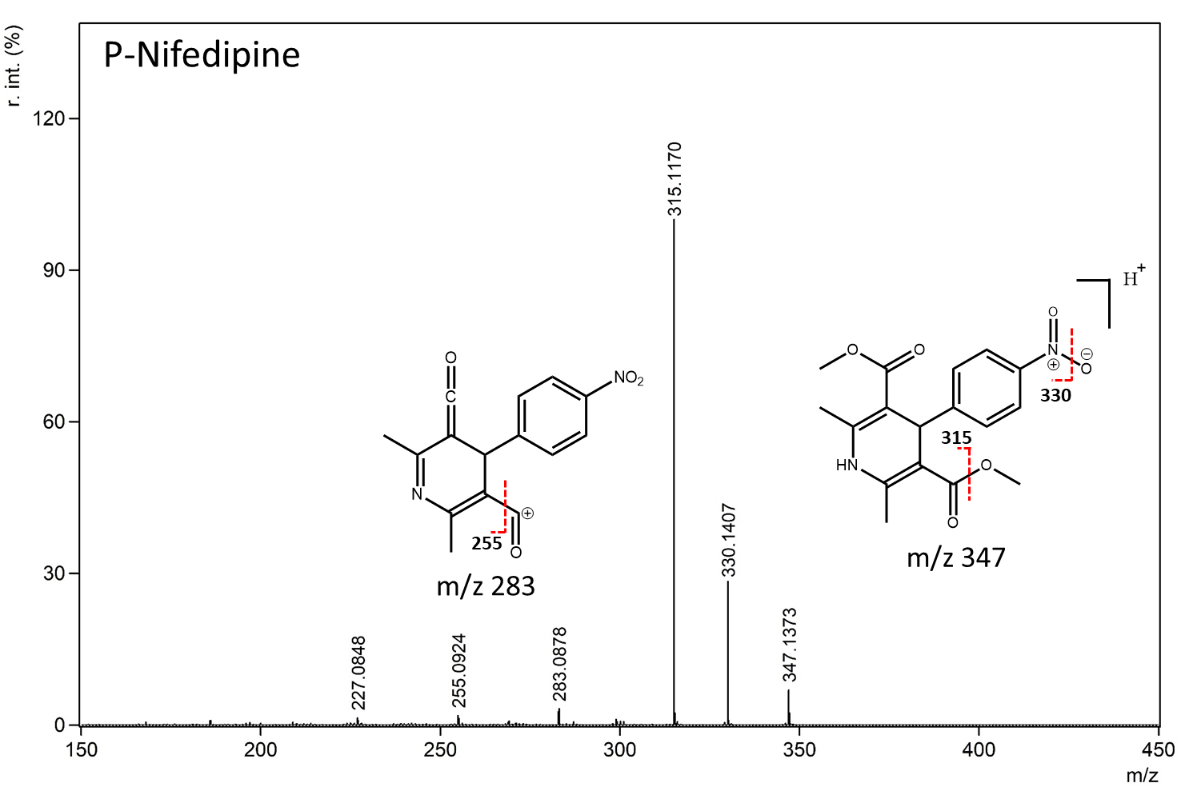


1. Figure S2: Fragmentation of protonated Nisoldipine (a) and Aranidipine (b) at 10 eV collision energy with proposed structures of fragments.


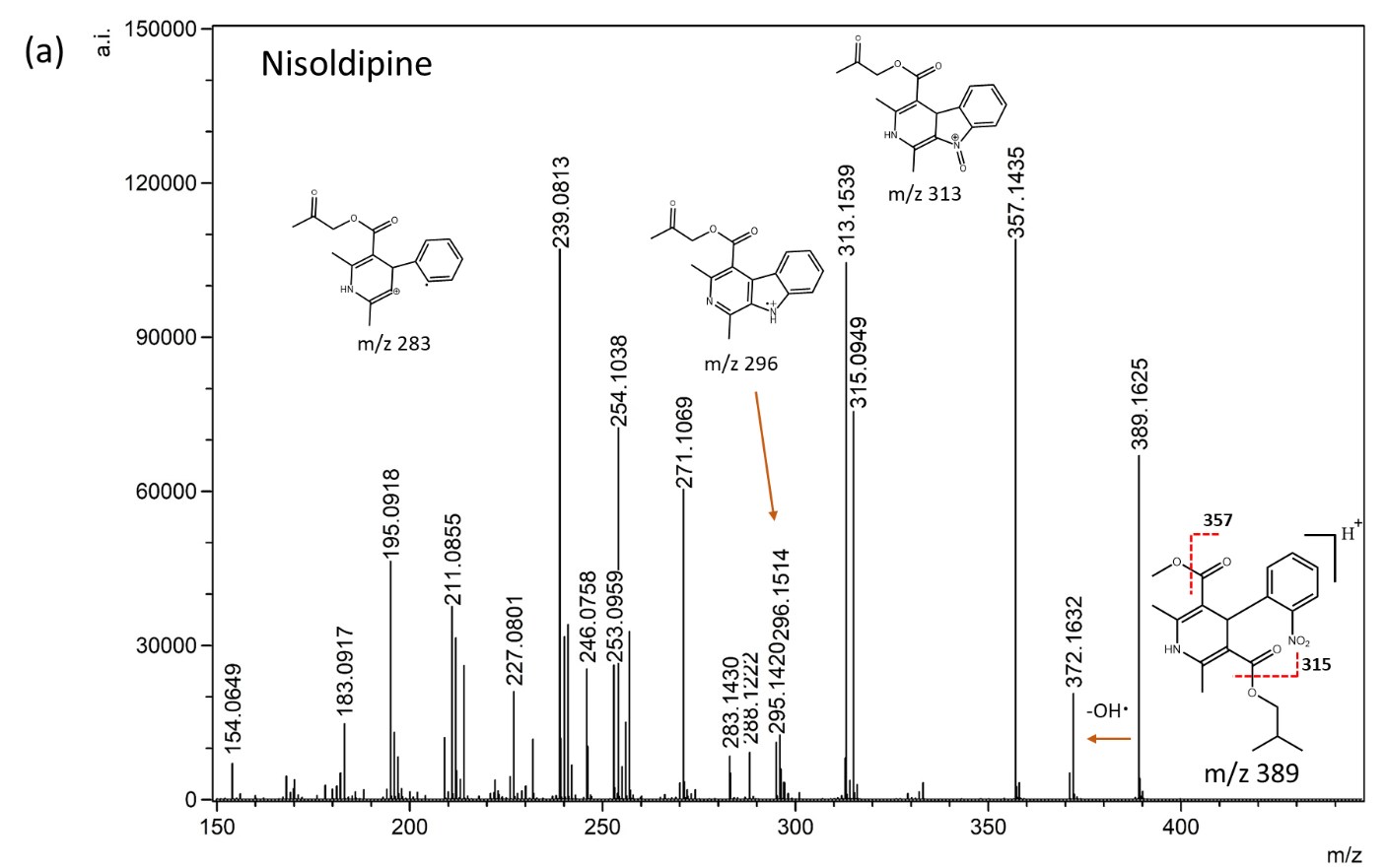


The m/z 313, m/z 296, m/z 295, m/z 288 and m/z 283 have similar structures and fragmentation mechanisms with m/z 271, m/z 254, m/z 253, m/z 246 and m/z 241, respectively, which only have the difference due to the side chains of asymmetric ester groups.


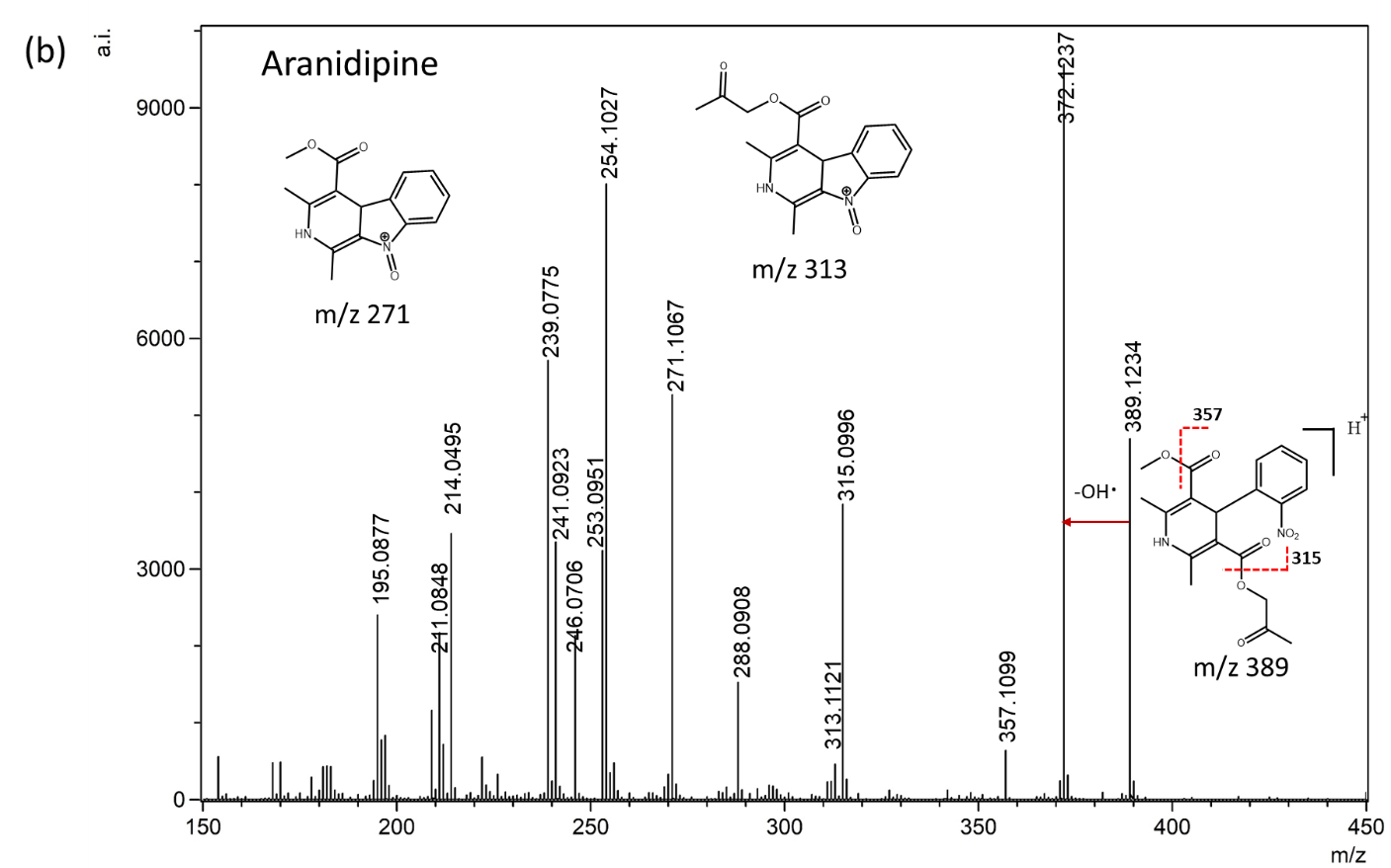


The fragmentation of aranidipine has higher preference on the loss of OH•. Unlike nisoldipine, the m/z 315 of aranidipine has higher priority than m/z 357. Therefore, the intensity of m/z 313 is also less than it in nisoldipine, which make other secondary fragments generated from m/z 313 less visible.

1. Table S2: lists of observed fragments for protonated Nifedipine (d_6_), Nisoldipine (d_6_), and Aranidipine

| Nifedipine (d_6_) | Aranidipine | Nisoldipine (d_6_) |
| --- | --- | --- |
| 330 (347-OH•) [336],  329(347-H_2_O) [335],  315 (347-MeOH) [318],  284(329-CH_3_NO) [287]  271 (315-CO_2_) [274],  254 (271-OH•) [257],  253 (271-H_2_O) [256],  246 [249]  241 (271-NO) [244],  239 (271-MeOH),  214 (246-MeOH)  211 (239-CO),  195 (239-CO_2_) | 372 (389-OH•)  357 (389-MeOH)  315 (389-C_3_H_6_O_2_),  288 (similar structure as 246)  271 (315-CO_2_),  254 (271-OH•),  253 (271-H_2_O),  246  241 (271-NO),  239 (271-MeOH),  214 (246-MeOH or 288-C_3_H_6_O_2_)  211 (239-CO),  195 (239-CO_2_) | 372 (389-OH•) [378]  357 (389-MeOH) [363]  315 (389-C_4_H_10_O),  313 (357-CO_2_) [319]  296 (313-OH•) [302]  295 (313-H_2_O) [301]  288 [294] (similar structure as 246)  283 (313-NO) [289]  271 (315-CO_2_),  254 (271-OH•),  253 (271-H_2_O),  246  241 (271-NO),  239 (271-MeOH),  214 (246-MeOH or 288-C_4_H_10_O)  211 (239-CO),  195 (239-CO_2_) |

The m/z value of fragments resulted from isotope label are indicated in bracket

1. Figure S3: CID-IMS-CID analysis of m/z 246.


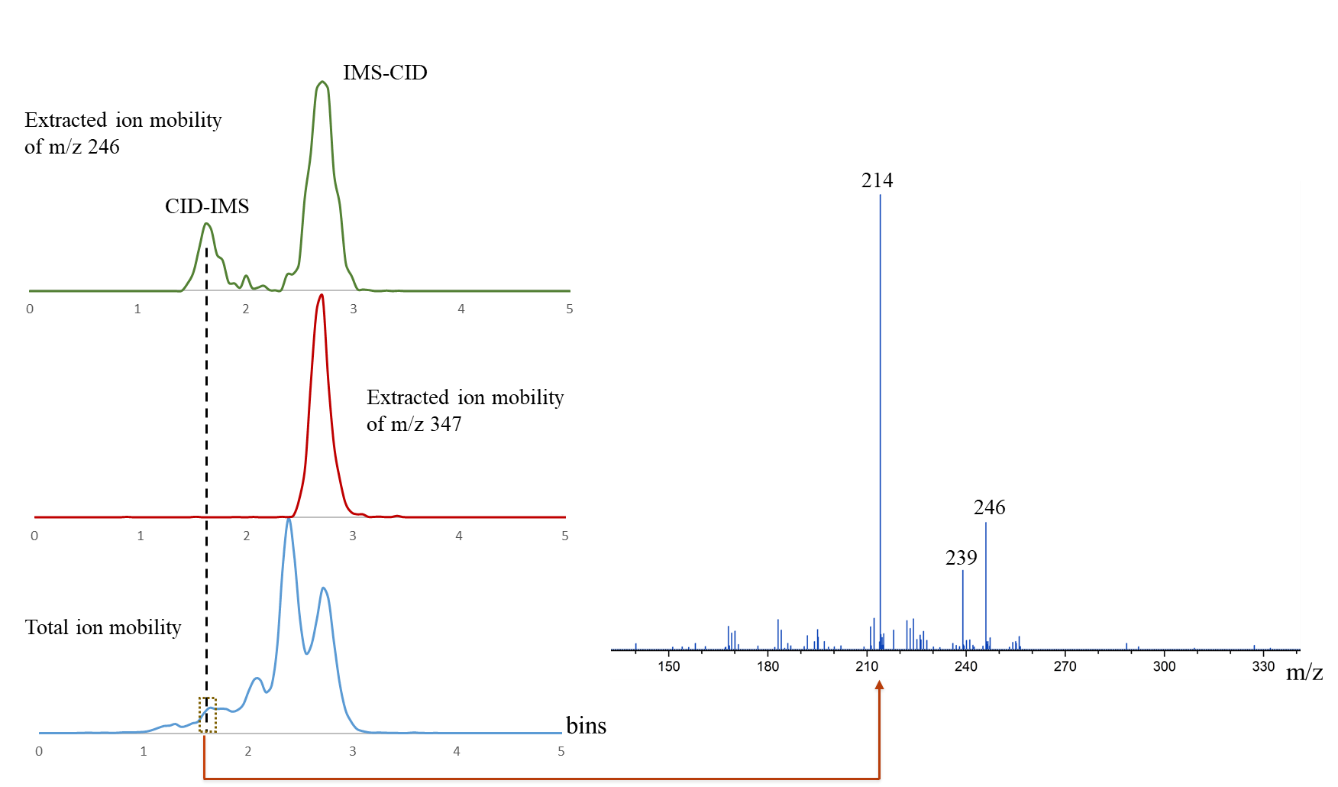


Ion m/z 246 is assigned as the direct fragment from precursor ion m/z 347. Ion m/z 214 is generated from m/z 246 by losing MeOH (proved by mass shift of deuterium labelling and nifedipine analogues). For the structure of ion m/z 246, the dihydropyridine ring may break by half but detailed structure and fragmentation mechanism are not clear and request further research to prove. The ion m/z 239 is due to the insufficient separation of ion mobility of m/z 246.

1. Figure S4: Precursor ion scan of fragments of protonated Nifedipine: (a) m/z 271; (b) m/z 254; (c) m/z 253; (d) m/z 241; (e) m/z 239; (f) m/z 211


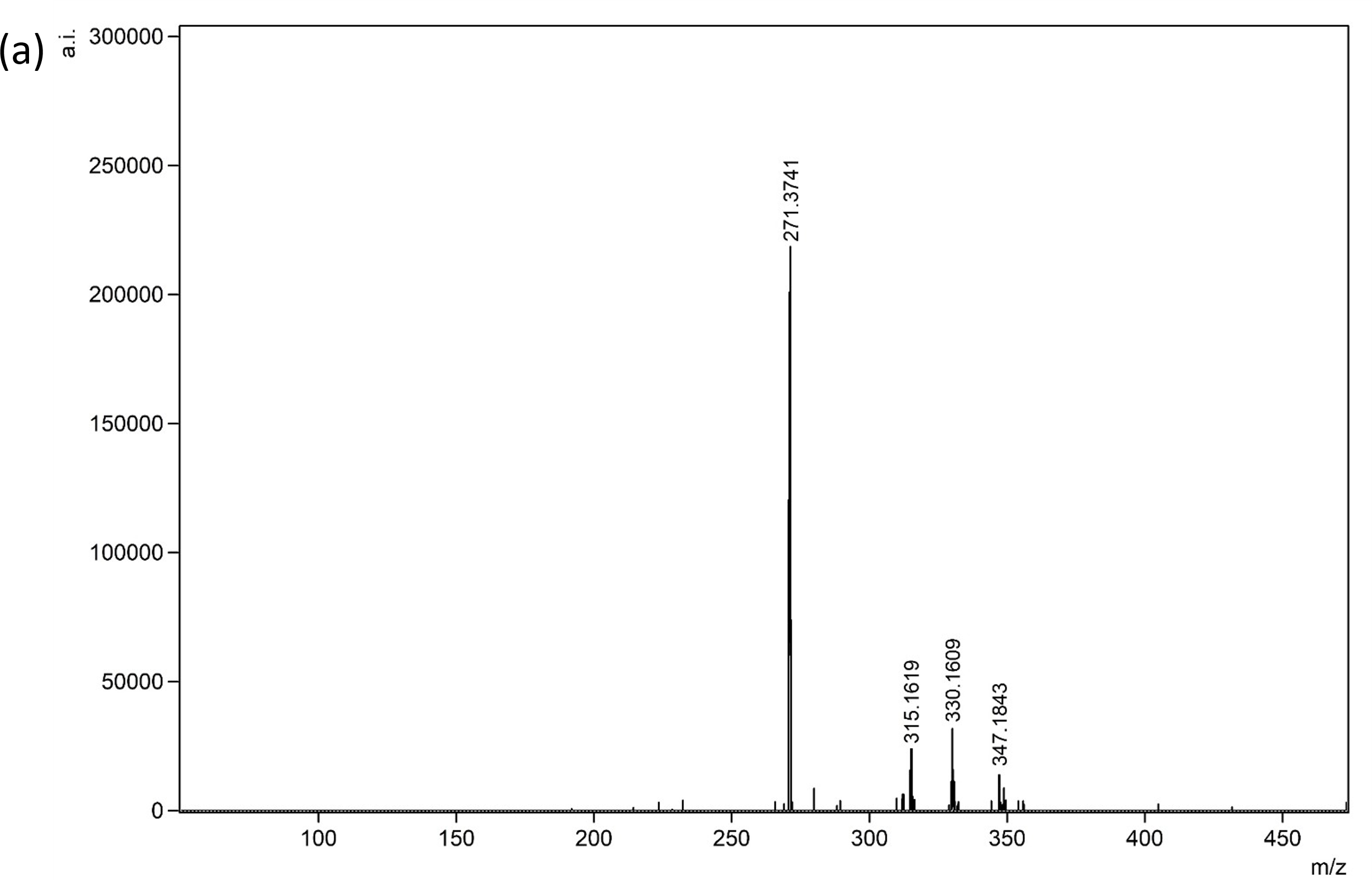


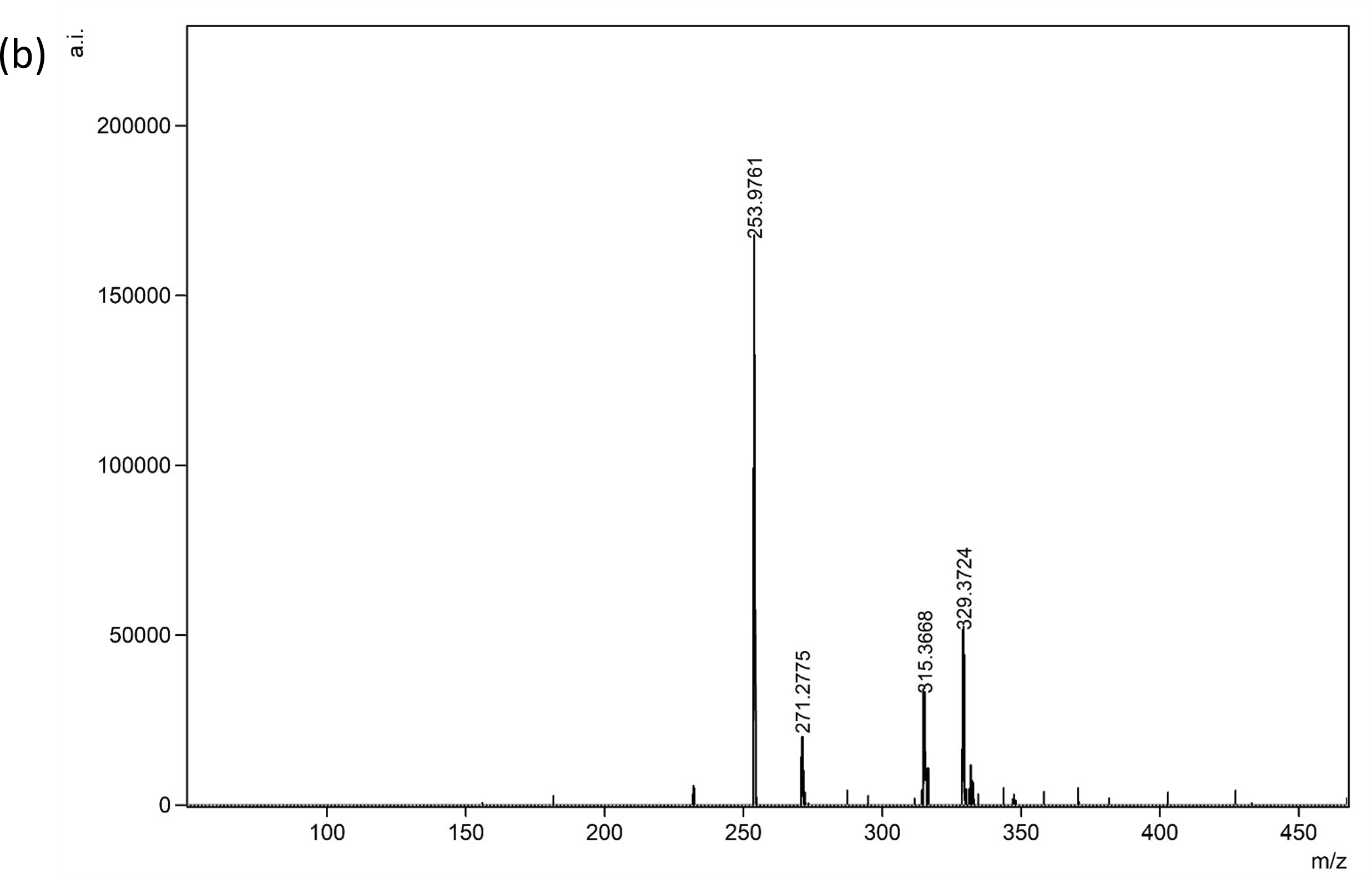


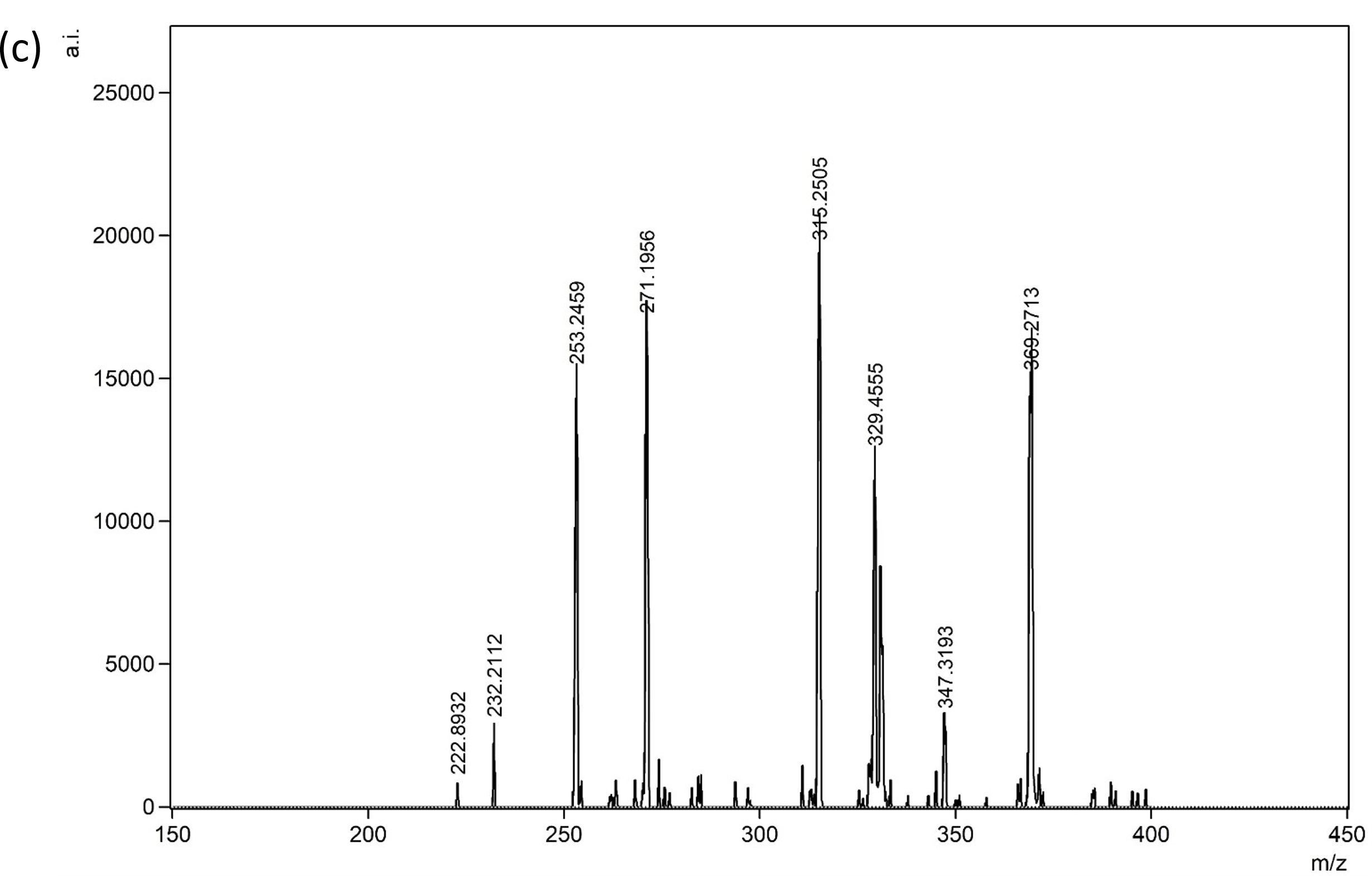


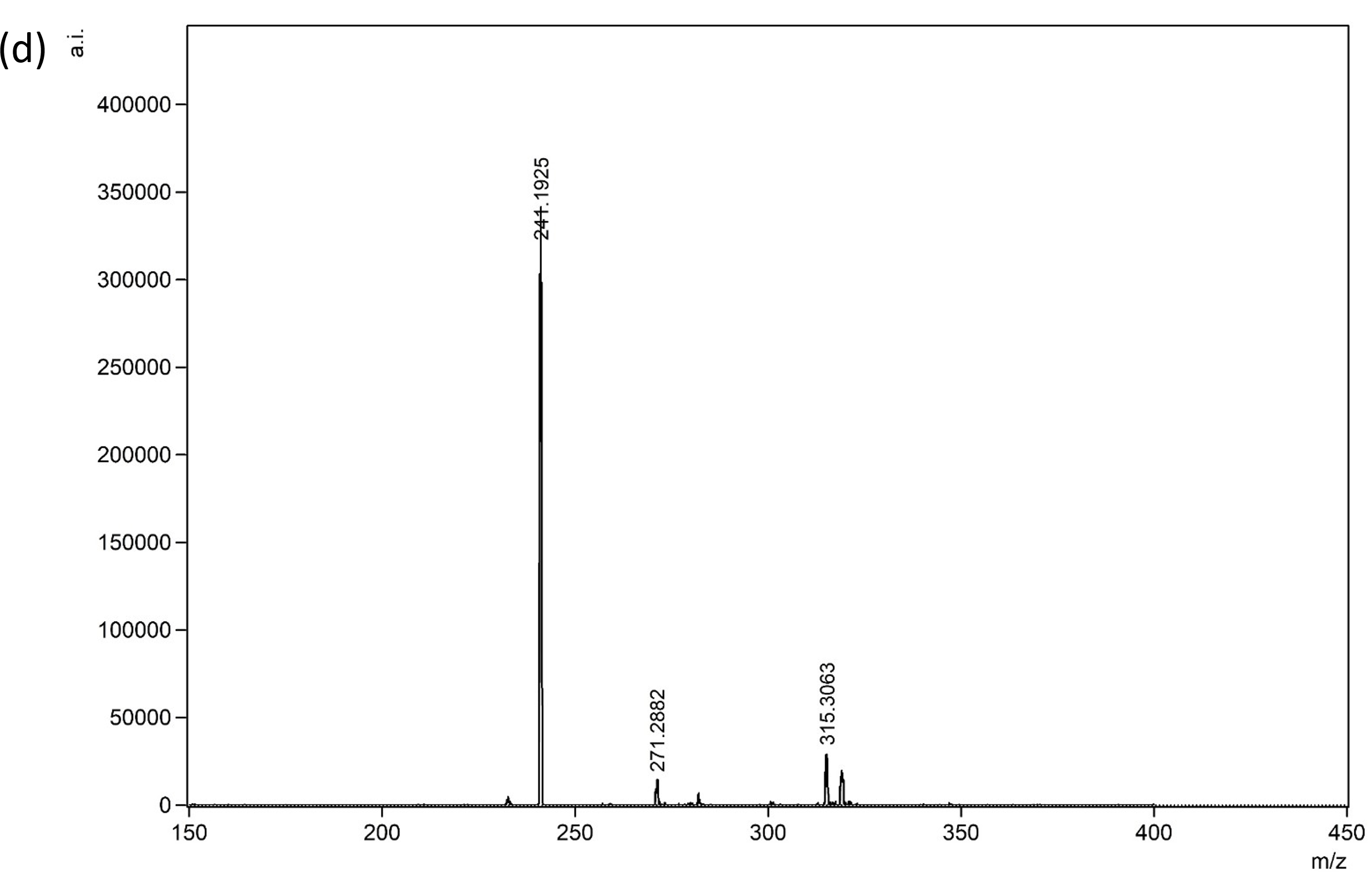


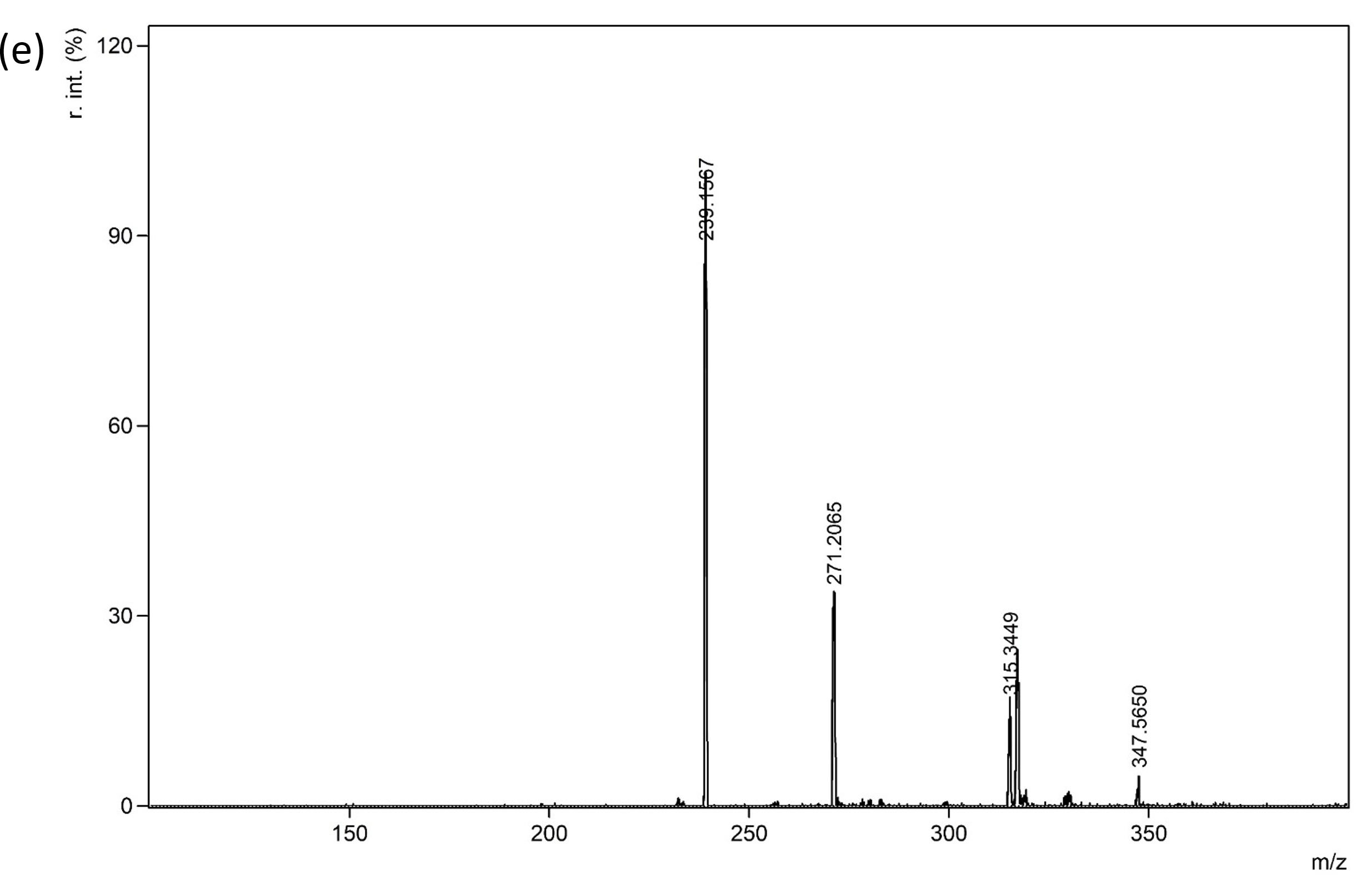


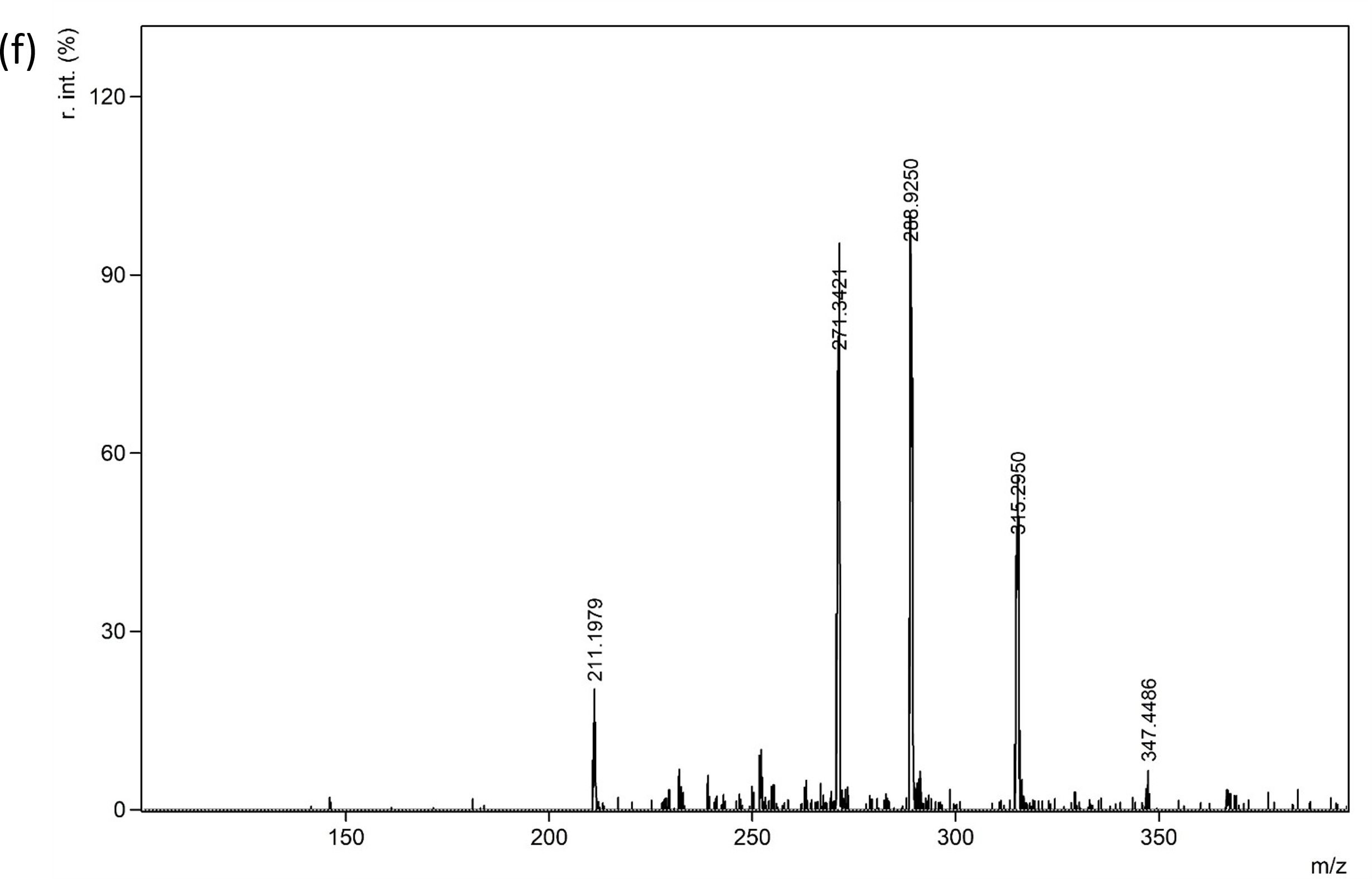


1. Figure S5: MS/MS fragmentation of sodium adduct of (a) Nifedipine-d_6_, (b) Aranidipine, (c) Nisoldipine and (d) Nisoldipine-d_6_ at 15eV collision energy with proposed structures of fragments.


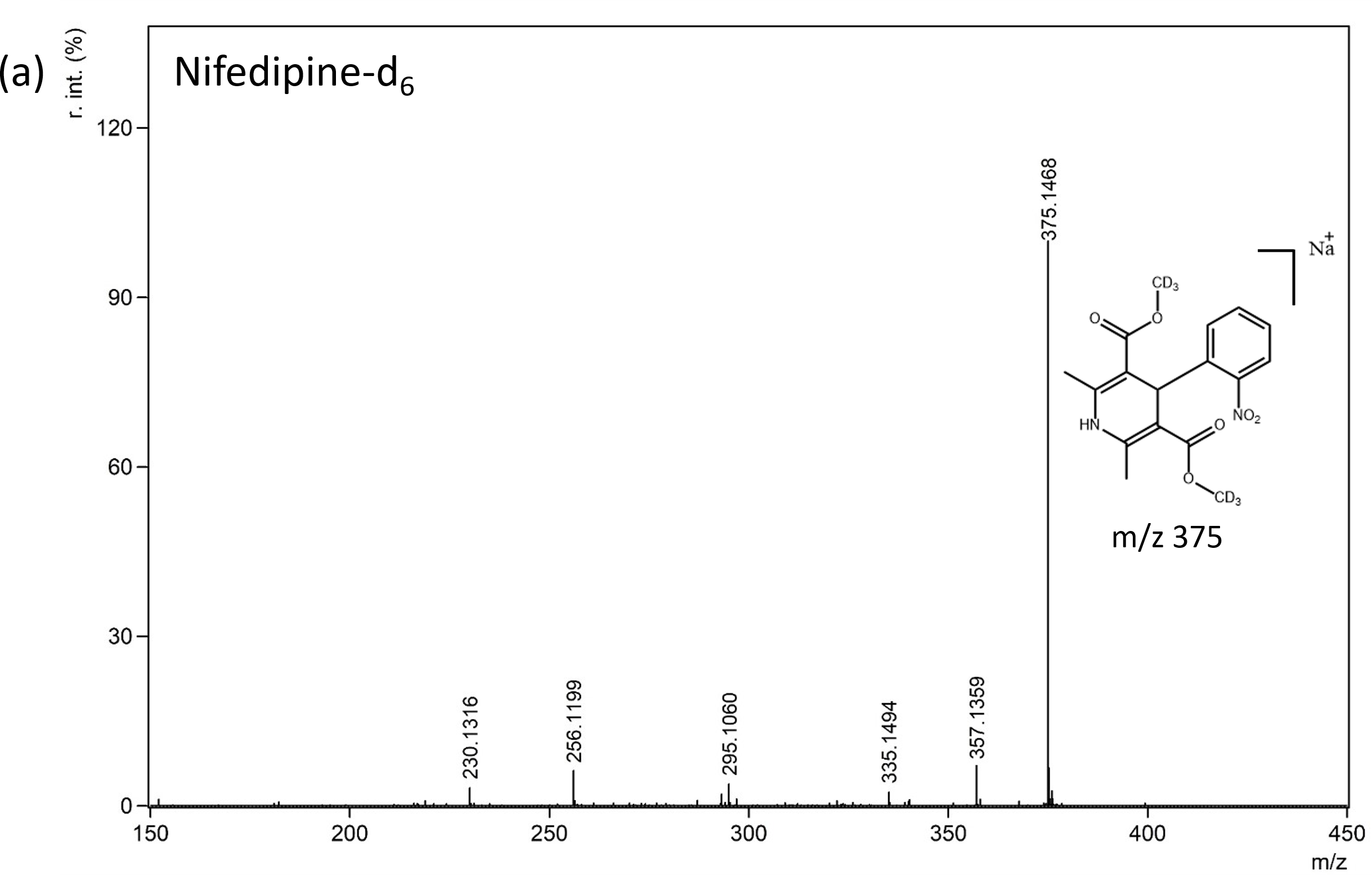


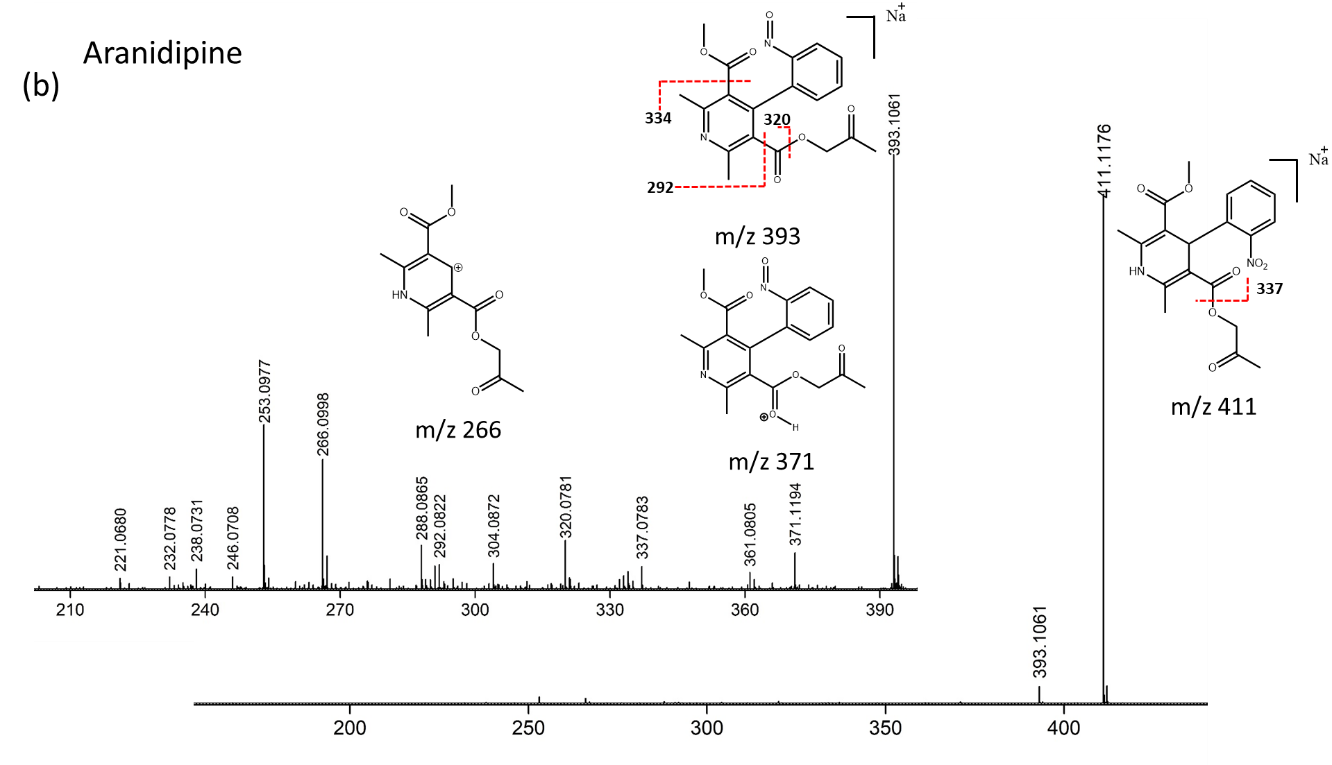


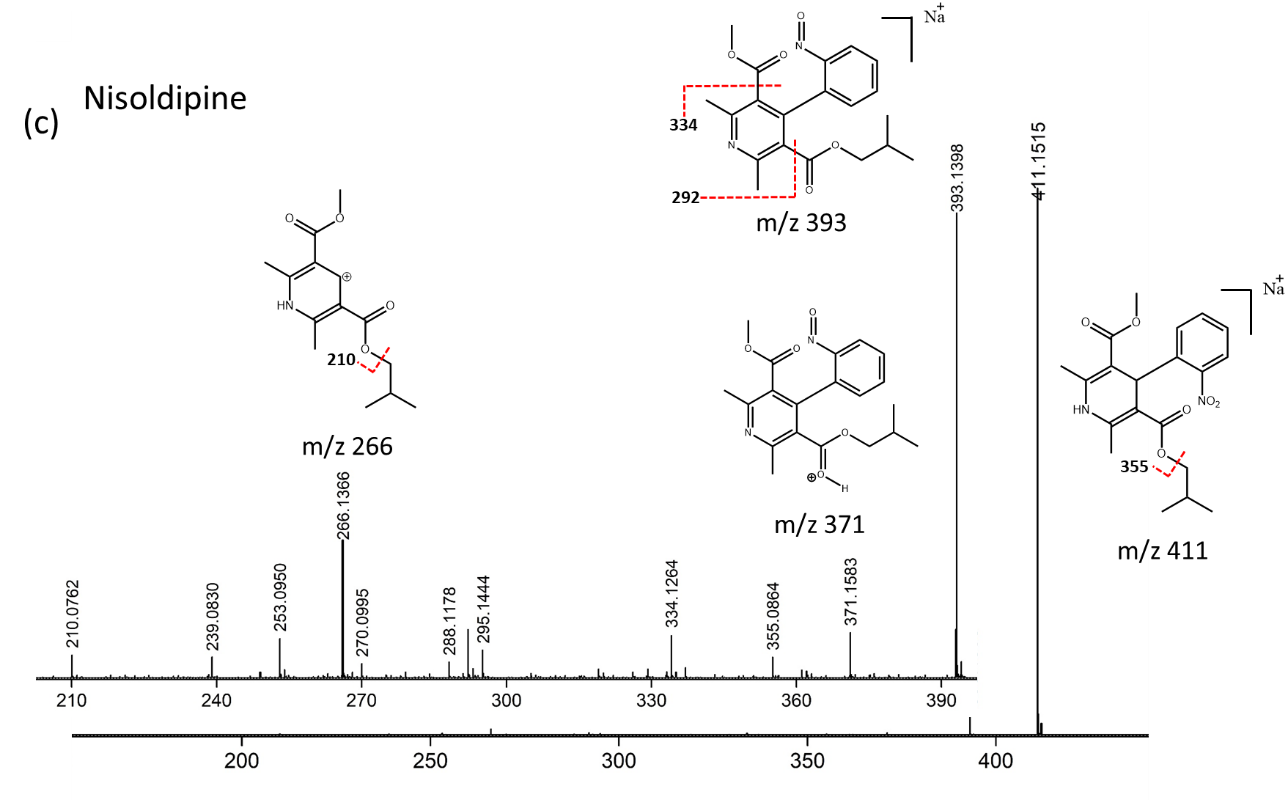

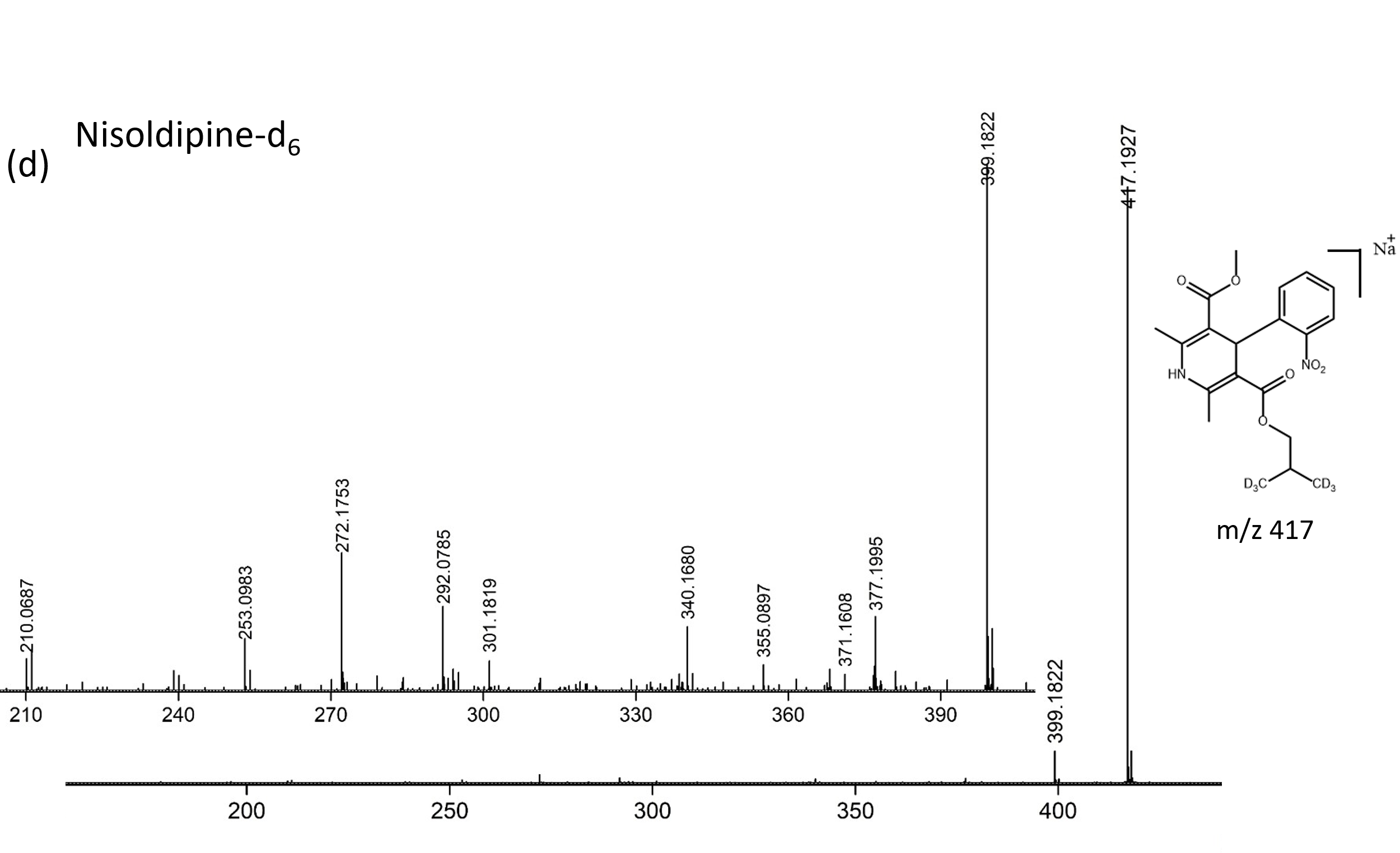


1. Figure S6: Extracted ion mobility of sodium adduct fragments of Nifedipine in analysis of CID-IMS-CID


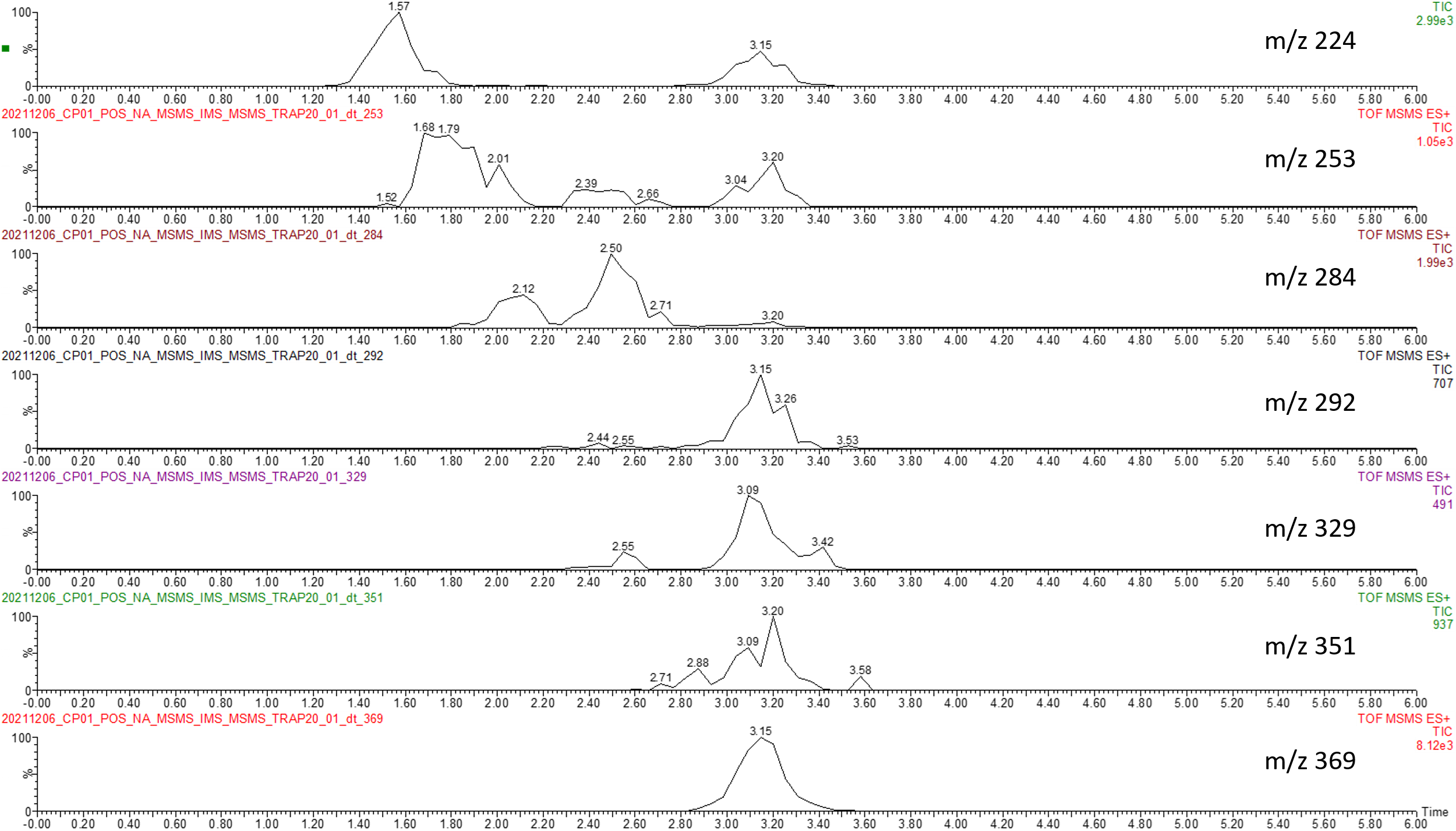


1. Figure S7: Sodium adduct of nifedipine after proton transfers, where grey atom is carbon, red atom is oxygen, white atom is hydrogen, blue atom is nitrogen and purple atom is sodium. Sodium cation is stabilized by oxygen atoms (dash line).


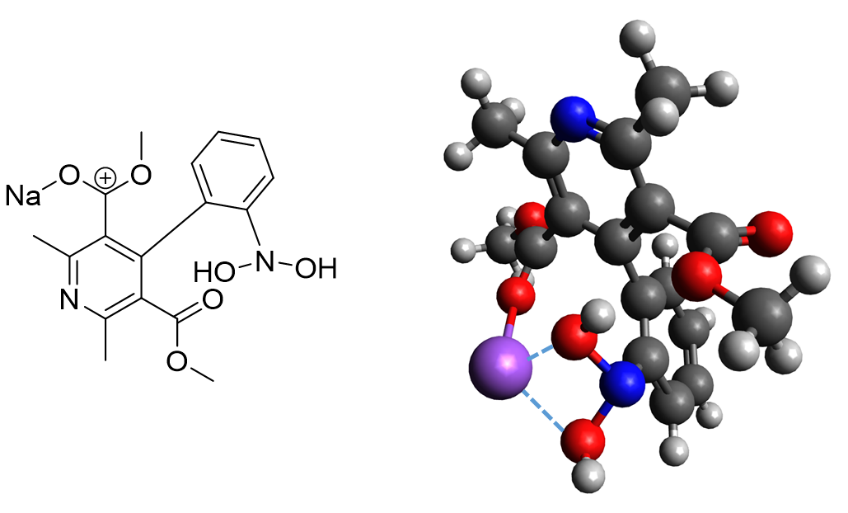


Appendix 2: theoretical calculation

Geometry optimization is performed by DFT calculation using ORCA 5.0.1 with B3LYP function and Def-TZVP basis set. Frequency check is also applied after geometry optimization to make sure the global minimum is obtained. Transition state is found by NEB-TS with B3LYP and Def-SVP. The found structure is further optimized by Def-TZVP (OptTS). The coordinates of nifedipine, m-nifedipine, p-nifedipine and related fragments are listed below.

Nifedipine [M+H]^+^

C -0.21936866847017 1.44080497385473 -0.35995443644644

C 1.03784746898446 0.96766457449420 -0.69923377984598

N 1.22059454417065 -0.36373120386680 -0.79039036733521

C 0.29469448615616 -1.34552040848459 -0.41873318518600

C -0.95616719345542 -0.96015449126441 -0.10909377369817

C -1.40189609171592 0.48382658061481 -0.24931238921646

C 2.26069534255170 1.78492450633746 -0.97803887829046

C 0.85139664136032 -2.73502487867230 -0.39000127415469

C -2.33406535408943 0.61647370197724 -1.46033977337363

C -1.82299047204294 0.33784557152051 -2.72700166478204

C -2.62220867520863 0.35378534867614 -3.86123571776323

C -3.98236948251283 0.62539797622050 -3.75668117494529

C -4.53027049925457 0.88413969359783 -2.51271712054348

C -3.70102834663273 0.89749157027766 -1.39634313876091

C -1.95002652206667 -1.95115230471142 0.36284815006897

C -0.47843724266650 2.81457572237256 -0.14675534814140

O -1.76680700880355 -3.14091334644061 0.44660808073363

O -3.10911405564698 -1.35333490686037 0.70300007623330

C -4.14890528043899 -2.22546593625121 1.19726735590184

O -1.63268050108001 3.29522439526644 0.17729214671615

O 0.47616804646856 3.69574981726350 -0.27295352563670

C 0.19014114754435 5.10309210494949 -0.03476736983674

N -4.35516647568762 1.21832682174200 -0.12656488876381

O -5.45716111005844 0.78121791029432 0.08654125726645

O -3.75517600304647 1.95914140066980 0.67496983838104

H 2.14488179767806 -0.68827090517185 -1.02783095387463

H -1.95749479823825 0.71616812263653 0.65236230041118

H 2.07085852264061 2.52417404015485 -1.75377521854810

H 3.08332772206829 1.14514434714843 -1.29494086718328

H 2.56993356835764 2.32939347880118 -0.08602813848311

H 1.88711101818450 -2.74452615486687 -0.73291326546886

H 0.26261230307470 -3.40375621715370 -1.01463986257077

H 0.81265261519638 -3.14690622596331 0.61798227289014

H -0.77575789177268 0.08376519388863 -2.82337941371696

H -2.18541812082771 0.14096817931447 -4.82781455591953

H -4.61211766392022 0.63213044099969 -4.63534129256024

H -5.58422833045305 1.08922690026655 -2.39277202678017

H -4.98756389539165 -1.57442064525125 1.42285925331464

H -3.80612967427287 -2.74397533457376 2.09044978750820

H -4.41632809495382 -2.95444526243194 0.43463765221371

H -0.15127933403360 5.24094814950711 0.98815161890080

H -0.56421880948678 5.44981927590540 -0.73667694810056

H 1.13757023072944 5.60384212223304 -0.20084025158853

H -2.41436985893713 2.66160530097924 0.29969081097546

m-nifedipine [M+H]^+^

C 0.83205115035425 0.59128567816711 -1.59718600066429

C -0.27369651524647 -0.24933706996849 -1.52910225266568

C -0.41506690793003 -1.15819542272725 -0.48304626241927

C 0.57127901345761 -1.21895515309354 0.49395936779252

C 1.66408097597303 -0.37446416394673 0.41247510442855

C 1.81689587494257 0.53922111730185 -0.61888320639980

C -1.63076876733170 -2.07538412455670 -0.38343755190082

C -2.38114740450635 -1.81381681123987 0.92029097082594

C -2.22631269374493 -2.67387297527733 2.00976844044108

N -1.55841198329965 -3.81309586271960 1.81334437005068

C -1.11497352715731 -4.31934774841431 0.57666050177861

C -1.21609625850743 -3.52871147113970 -0.50599979467554

C -2.72962829706951 -2.45078860576776 3.40030389055092

C -0.59935128310510 -5.72242975828487 0.65092861093399

C -0.86638529644533 -4.05193885349331 -1.85086051802116

O -0.50175378623265 -5.17806113091975 -2.08786645721231

O -1.02298098354041 -3.10821360576225 -2.79611627140166

C -3.12714961666737 -0.64314384818530 0.99172798311183

O -3.71991404844640 -0.24993885003394 2.10584606764723

O -3.22276288645949 0.08025459138572 -0.09863065467976

C -3.88688101511804 1.35940317771046 -0.10442853090189

C -0.71113206012888 -3.51438179822708 -4.14511835521420

N 2.69255378747583 -0.45960728227043 1.47117820253372

O 3.64709718136757 0.28989892671735 1.39076338664679

O 2.51403007204113 -1.28142981298076 2.35882774889276

H 0.92991980976977 1.28947660331997 -2.41756945543002

H -1.02891277854399 -0.21145747964767 -2.30311529894779

H 0.51786577433911 -1.91338308633204 1.31862952427114

H 2.68550932353840 1.17975223790181 -0.64718594296928

H -2.28568517037776 -1.84153819419172 -1.21907490330592

H -1.43481254493030 -4.42055653840816 2.60982010679299

H -3.81827652261102 -2.39504836417094 3.41087862336909

H -2.35535438695135 -1.51085695929480 3.80387065039886

H -2.41842474827683 -3.26184900983195 4.05677221755483

H -0.39968803014565 -6.00300727995680 1.68683653252867

H 0.31150073692062 -5.83392254223766 0.07014564299692

H -1.32202761703626 -6.42566478821051 0.23382195634633

H -3.75998608273400 1.73771116690794 -1.11295720556183

H -3.40903428922902 2.03998296086261 0.60178976825691

H -4.95284909752699 1.24148348265299 0.10154403494785

H -0.90097803685149 -2.64116863621086 -4.76155907735353

H -1.34673107523965 -4.34621653017868 -4.44279025527161

H 0.33264813048400 -3.81597299582231 -4.21227692124855

H -4.25028811927252 0.55212681057530 1.99541121314668

p-nifedipine [M+H]^+^

C 0.17555164612420 0.74764485460144 -1.13767141538509

C -0.79886216015078 -0.23905265275164 -1.07103452364355

C -0.68680899157018 -1.28703103242653 -0.16064716137629

C 0.41151249559108 -1.32832101303021 0.70006845449972

C 1.39209826588370 -0.35101514114207 0.64914496711324

C 1.25955461806940 0.67160118701957 -0.27868206034333

C -1.75673945552491 -2.37196848786826 -0.10228152036393

N 2.30480211397670 1.71545025565104 -0.34704342314894

C -2.45999311680557 -2.34430338845799 1.23944804316627

C -2.17780987308314 -3.25238796005178 2.18512255797927

N -1.26990460332132 -4.28817961017693 1.85785134984374

C -0.84015568835722 -4.60581926273055 0.63420238346223

C -1.17190765879786 -3.74065927009209 -0.40997095297982

C -0.04259344809072 -5.86670680397117 0.53166915730621

C -2.68564113612118 -3.37253820308839 3.58545975839966

C -3.44284072512155 -1.22626022514038 1.33969282509804

C -0.94153166058756 -4.05126733158563 -1.74477347899296

O -0.40550089897688 -5.20291572759001 -2.11042838296916

O -1.29002729009512 -3.16756823059624 -2.64882815262863

C -1.15449129932173 -3.43584579133634 -4.06086608670584

O -3.78353877799715 -0.87982838260718 2.58382300165458

O -3.86633649132803 -0.67568499789067 0.35036335052766

C -4.72940946474978 0.21034470793100 2.69514170714961

O 3.21584860787531 1.64819992638948 0.46055729198731

O 2.18815127340702 2.56981440029291 -1.20856367337794

H 0.10579633906415 1.56876906314852 -1.83540331613326

H -1.66386236869627 -0.18543881061730 -1.71839807898304

H 0.50442529531931 -2.12458001898048 1.42744504423594

H 2.24647342060558 -0.36612346973601 1.30903791493351

H -2.50426411730133 -2.12956714013921 -0.85337587583974

H -1.03001013804904 -4.91810861475500 2.60926380961558

H 0.18727352531335 -6.25767008594422 1.52203162012784

H 0.88971008931448 -5.69467775972190 -0.00328721095439

H -0.59670931305637 -6.62727391115539 -0.01960388826663

H -1.89873986789177 -3.09483869769215 4.29364381165693

H -2.97702114015961 -4.40464651969655 3.79596503229276

H -3.53237692305660 -2.72190661597807 3.75760805052244

H -1.55506149611855 -2.55676182015359 -4.55371554827818

H -1.74203391233301 -4.31103632421834 -4.34249854346175

H -0.10201093834583 -3.54825923786098 -4.32913544484789

H -4.32260921421620 1.10387894988438 2.22643750524417

H -4.86862899490053 0.36053797842305 3.76100634919448

H -5.66733622432302 -0.05820416084758 2.21308534010000

H -0.24979030209541 -5.26698462331038 -3.06354058743086

Transition state of formation of m/z 315 from nifedipine [M+H]^+^

C 0.91241436411868 1.06417453737944 0.09440804147619

C 2.21312135269827 0.65267144906508 -0.19701788717314

N 2.49411902748692 -0.64834643668004 -0.10542821616332

C 1.63618006684427 -1.65796496102361 0.39014750312302

C 0.34853602141233 -1.34745108731769 0.61426303577382

C -0.21528990504185 0.03684652423024 0.30027074818253

C 3.35269423003351 1.51018472913120 -0.66654982339651

C 2.35553023026452 -2.95391495986043 0.59885844315889

C -1.16602454162799 -0.02578875650525 -0.90372216757488

C -0.63841210676868 -0.30022929962078 -2.16737418462646

C -1.43892558909872 -0.40567990861207 -3.29634320599770

C -2.81617833026775 -0.26136468928789 -3.18526013194928

C -3.37745521120178 -0.02142363259982 -1.94139854431737

C -2.55796438191783 0.10669484630442 -0.82606999148678

C -0.59404151648716 -2.36754399040597 1.17885831854206

C 0.51205353189691 2.39312433343358 0.12565553526267

O -0.41967205806575 -3.56106293573744 1.17105594472255

O -1.67396762552547 -1.77776674952580 1.70602600646166

C -2.66166909829447 -2.65524927990518 2.29952713227040

O -0.60177400241623 2.94377808186372 0.07834674501060

O 1.38654546455344 3.57709872821138 0.22739090345829

C 2.10988158561557 3.83353009526143 1.48610225988184

N -3.26318263018716 0.37926764336269 0.44816908796826

O -4.32245344986904 -0.19625200726970 0.62128294775805

O -2.76308202248239 1.16955179915492 1.23404778467208

H 3.42684692308269 -0.94568423375514 -0.35482476814812

H -0.79194932755206 0.36132946800088 1.15963989353218

H 3.03013911503160 2.48018694873596 -1.02694958599380

H 3.87196990047789 1.00657625761059 -1.48431903571244

H 4.08287478075606 1.65184150935531 0.13481098325998

H 3.27758778066491 -2.78523106337695 1.16248466207895

H 2.62846634498132 -3.38988325943539 -0.36781883544601

H 1.73641247011628 -3.67328169677512 1.11963817683158

H 0.43008000125980 -0.44329537278619 -2.27226705138699

H -0.98800007358633 -0.60949950740976 -4.25884112447830

H -3.45144786470474 -0.34366401767747 -4.05683631907437

H -4.44690475667385 0.07266503091575 -1.81919670406068

H -3.46212997317910 -2.00132709946029 2.62981637807449

H -2.22155210604512 -3.19243053188676 3.13767302168427

H -3.02124828837674 -3.36593208490076 1.55815057976369

H 1.42411471651136 3.72160474870717 2.32409420397454

H 2.47275622246079 4.85350452035742 1.40377319199823

H 2.93803373455729 3.13736146654848 1.55696276036542

H 0.25296699454583 3.97827484418583 0.16876328769991

Transition state of formation of m/z 315 from m-nifedipine [M+H]^+^

C 2.40043191415860 1.91804580472143 -1.68847864592927

C 1.23393289277739 1.16611088700342 -1.61353734475418

C 0.71240579785873 0.76128806115548 -0.37830852428306

C 1.38887791658155 1.13705436461021 0.77819160929094

C 2.55301908108801 1.88564118737587 0.68321141479639

C 3.07943920348585 2.28680644803693 -0.53277149342370

C -0.55958245570926 -0.10260985284454 -0.32802120846782

C -1.30036970325752 -0.00800543972046 0.99706397753877

C -0.96680592174261 -0.89753662809860 2.03337757510771

N -0.20993311025087 -1.94971331168602 1.71793053573745

C 0.04451008312928 -2.40704343541960 0.40749281408762

C -0.21486396498842 -1.56501830097343 -0.61250899446466

C -1.40498371581199 -0.78133169404711 3.45640717651353

C 0.52911395416666 -3.82445763078347 0.34292152706878

C -0.14707818049458 -2.06351283469386 -2.01228395920691

O 0.28031064033994 -3.14354149730893 -2.33770116634050

O -0.65297416089961 -1.17425424486342 -2.89000322970304

C -2.29955960629600 0.91733345687432 1.23374045619416

O -3.08906008709073 1.15413140916116 2.17910684207002

O -2.77977967912111 1.87171842106081 0.24556410523743

C -1.95888343351974 2.99227387578696 -0.25302468496351

C -0.68143864167248 -1.60603655955110 -4.27040903043511

N 3.24274675533768 2.27142940122677 1.93817447557087

O 4.26349119952821 2.92421139346461 1.83930798361842

O 2.73646228257364 1.90829962014128 2.99027345311869

H 2.78412574299213 2.22032637397898 -2.65397700162285

H 0.72338877958589 0.88374367288842 -2.52480475305417

H 1.03910217498697 0.87572661974427 1.76472003535713

H 3.98698371922306 2.87161171974453 -0.56356090446011

H -1.22223820951346 0.22914883903830 -1.12530677549893

H 0.05119130294262 -2.57612319453755 2.46671530094681

H -2.46730149300788 -1.02402880329725 3.53970956099173

H -1.28390951388595 0.23438141371882 3.82980897334110

H -0.84319355572455 -1.46469357878108 4.09253577487294

H 0.69500924475887 -4.22344425216833 1.34513576735578

H 1.45463357049548 -3.89547454621426 -0.22475702876467

H -0.19405766878597 -4.46160518234475 -0.16689199376452

H -1.25341454329586 2.60442966095648 -0.97812992965389

H -1.45064595074536 3.46760870516442 0.58345990451096

H -2.66817639165320 3.66611556128691 -0.72349173610654

H -1.12761544132397 -0.78369826424434 -4.82160459603848

H -1.28353819486725 -2.50746220599464 -4.36804151616501

H 0.32893120457702 -1.80749715195988 -4.62189549946792

H -3.46870383692921 2.01965171239222 1.22466075324161

Fragment m/z 315 of nifedipine

C -0.38473586375837 1.35736134177570 -0.36758159435378

C 0.94175794271222 0.99689810256372 -0.62893149606063

N 1.24393795665453 -0.29615464352084 -0.66026941190968

C 0.36431231471889 -1.35202116777991 -0.33210927942359

C -0.93137469039302 -1.06726915019250 -0.11929483068862

C -1.50907789371978 0.32608165307873 -0.27865462561976

C 2.04570732139340 1.98028351487936 -0.84578388533056

C 1.03568553945095 -2.68703947623152 -0.24515951800453

C -2.43126681339331 0.42054734866795 -1.49209294999892

C -2.20385371222641 -0.37880586357071 -2.60741149516896

C -3.04994081278705 -0.35024139017696 -3.70953779154338

C -4.16983880026747 0.47211212024671 -3.71383311566764

C -4.42944885962085 1.27640223412368 -2.61637502015954

C -3.55607011461070 1.25287683569354 -1.53675697999509

C -1.87406071143475 -2.15097462419770 0.27238463242032

C -0.59910263588613 2.69712458680646 -0.26572305413188

O -1.58817028359992 -3.32057367861563 0.34847447494062

O -3.09452425744219 -1.65725250680967 0.52358701329955

C -4.10189761827978 -2.62056294942485 0.90208134800537

O -0.54397466493639 3.83320870693839 -0.22199595409278

N -3.85804679172966 2.16329465987757 -0.43168487099406

O -2.90114191139055 2.59756612281758 0.22848115226110

O -5.00646571467982 2.47085195906052 -0.22027032205136

H -2.09246352897218 0.53418789888291 0.61212468845996

H 1.77409732270580 2.71418484927042 -1.60471548990609

H 2.96186029895056 1.48348856462503 -1.16009831666988

H 2.25275021877288 2.52076007253993 0.08116491575470

H 2.09191784442710 -2.60746327592953 -0.50724032773084

H 0.55911592476632 -3.40752379118712 -0.90688740809119

H 0.95575542908994 -3.09863300074326 0.76044050432884

H -1.35942150750451 -1.05461653396705 -2.60772858185315

H -2.83942733007467 -0.98250772745156 -4.56160280980426

H -4.83591141101906 0.49139517112990 -4.56501449688632

H -5.29105923052444 1.92710272007469 -2.58302240985621

H -5.01420896416812 -2.04710470347982 1.03028459221631

H -3.81727816027205 -3.11104593275417 1.83110509510660

H -4.21592792214767 -3.36708468972844 0.11842227658803

H 2.20397209119630 -0.55121335729156 -0.84145465738867

Proton transfer of fragment m/z 315 of nifedipine for imagined MeOH loss

C -0.33590217383876 1.33528393358978 -0.36571832645532

C 0.99024903487039 0.92206603387418 -0.62465146927328

N 1.32631175605932 -0.35982041573669 -0.71508770237531

C 0.44507455433308 -1.33462366471722 -0.44136628540389

C -0.90472106967057 -1.07004775272550 -0.14193548562171

C -1.48119848223590 0.33153888101158 -0.29236906441724

C 2.10616134382232 1.89288219428685 -0.81720703245456

C 1.04549882063415 -2.70332224004829 -0.47873088039083

C -2.38473261306161 0.46044183503338 -1.51642419353199

C -2.02787625201405 -0.15138632070553 -2.71673243735399

C -2.82674122591319 -0.05296588552718 -3.84880169902006

C -4.02895933975414 0.64387548041464 -3.80069945327023

C -4.42306717995794 1.24490079498832 -2.61628100671838

C -3.59396701568580 1.16122368567483 -1.50578901016883

C -1.80615861113664 -2.03414798989961 0.29403295095801

C -0.56659622254466 2.66129638016805 -0.27064474664256

O -1.46095435351132 -3.29343514220308 0.49325069798409

O -3.04231996842056 -1.64215858074014 0.52253824449847

C -4.04987035648255 -2.53344107633744 1.04359973161088

O -0.67797496831208 3.79343892417159 -0.21066386769555

N -4.04990855223831 1.85106593330006 -0.29096632429090

O -3.19788937849924 2.41893167188921 0.39116897835434

O -5.23648837122516 1.83149205747618 -0.03513753328910

H -2.06390591399903 0.55027663675412 0.59480596646375

H 2.44056853072509 1.83472750612600 -1.85673011664850

H 2.95230569814162 1.59048383947864 -0.19986384388634

H 1.84138877257084 2.92372462871126 -0.59203287028380

H 2.05104640473316 -2.62690149961251 -0.88139015297682

H 0.45063909281942 -3.39346377216244 -1.07673471499699

H 1.09968642539429 -3.12507735898703 0.52770252225490

H -1.10607961168288 -0.71555523172431 -2.76723020211571

H -2.51109403352887 -0.52554553017447 -4.76935357659519

H -4.65599415601354 0.71949344313457 -4.67824806179534

H -5.35649402889478 1.78363536773114 -2.53951677370423

H -4.94267580643229 -1.92414850372411 1.13607777493083

H -3.75862891788478 -2.90664668292093 2.02663329242231

H -4.24156642019918 -3.34706402638553 0.34132631113632

H -2.20375541096570 -3.85476755348239 0.75754036076281

Fragment m/z 271 of nifedipine

C 1.30389874217789 0.18211139674976 0.77890585586626

C 1.04653718585297 1.23644712124650 -0.01672901055806

C -0.37964297044336 1.51925222431034 -0.44037737056156

C -1.17898298520822 0.24738024742953 -0.41048895827915

C -0.89670926566647 -0.81914202460366 0.42954063654393

N 0.27229330186254 -0.73996358150746 1.07207589404173

C -1.73770538086240 -2.03255039474194 0.58991480062259

C 2.59944062488706 -0.17664667795400 1.43410007154492

C -0.77196079396005 2.09034059200716 -1.79211185106671

C 2.12679455105278 2.21473779138135 -0.32335625787696

O 3.26058810095258 1.92271564503198 -0.59929595683926

O 1.66590237566122 3.47133371097478 -0.22626143020138

C 2.63530974391543 4.52001090036134 -0.46215291763360

C -0.39432451334159 3.22455544528748 -2.49202523712365

C -1.04414853682290 3.50813000030963 -3.69332611526129

C -2.08349297235867 2.70936780051698 -4.18333199595289

C -2.49310199014793 1.58424674437121 -3.48700854258568

C -1.81188529224757 1.31460774315083 -2.31033622083497

N -2.05475110005500 0.20375005808514 -1.45342537301368

O -2.90727094855503 -0.65979759536621 -1.67976928118786

H -0.79644528063525 2.22509095403243 0.29869802804644

H 0.51080891796726 -1.49223495508381 1.70352634852127

H -2.75275380892311 -1.76459356957349 0.88293548935546

H -1.30797341292797 -2.71749630049265 1.31965137466958

H -1.82746177667652 -2.54057552099507 -0.37402659049067

H 3.32065153759606 0.62961097111340 1.36896664174886

H 3.04061453884104 -1.05153171484140 0.94918051071718

H 2.43119044450321 -0.42143709852024 2.48598456217238

H 2.09732332389618 5.44976399037732 -0.30672843894412

H 3.01872673791094 4.45203300884052 -1.47904475609870

H 3.46081961221878 4.42850270500668 0.24056103451596

H 0.38357583772403 3.87351722355546 -2.12596095904498

H -0.73987353456429 4.37865762760621 -4.25942383996783

H -2.56904119020229 2.97408398682292 -5.11231694337930

H -3.29758982342135 0.94546154511096 -3.82231320146421

Fragment m/z 283 (MeOH loss) from m/z 315 in nifedipine [M+H]^+^

C -0.42800112235872 1.33979855821246 -0.24295211999337

C 0.87102993534275 0.96684680295989 -0.64504859716329

N 1.23510962360588 -0.31580311969832 -0.78086630449763

C 0.43212548399150 -1.29062964692694 -0.38452263939574

C -0.90826323777261 -1.03972330492314 0.00491180211821

C -1.56900349348640 0.32274726346130 -0.19337532759812

C 1.94718947071575 1.95322815803470 -0.93400588492701

C 1.01691531042783 -2.66078810333115 -0.36747494256117

C -2.41659002984654 0.38877991122822 -1.46417591358573

C -2.08967663716515 -0.39856018041946 -2.56643497448508

C -2.82943104197914 -0.35712669956820 -3.74188851029664

C -3.94546551462645 0.46331468960420 -3.83910524598297

C -4.31027881893900 1.24571734153178 -2.75605458946532

C -3.54271310906479 1.21486348141225 -1.59884561353247

C -1.62150106900674 -2.04147216013732 0.54402163652514

C -0.62276046867386 2.64751733923565 0.06115853180108

O -2.23720494779898 -2.88395570336620 1.00256446391604

O -0.63296767032635 3.75558653382117 0.31532747350436

N -3.97027109244875 2.11223911914592 -0.51628310950660

O -3.10474032066564 2.50242745906371 0.27663310536053

O -5.13246018704593 2.43839507433617 -0.46187807651670

H -2.19178506220579 0.53893485494835 0.66760390403002

H 1.66057154121209 2.98968209419420 -0.77638200759560

H 2.24974275442436 1.82341908949805 -1.97707188126398

H 2.82393926425906 1.71618988150674 -0.32908093630961

H 1.27047943125412 -2.94378308211647 -1.39276339015238

H 0.35094214205695 -3.41540533983496 0.04817704174279

H 1.95268960057853 -2.64988980189560 0.19251926692452

H -1.24194661698974 -1.06638668247977 -2.51235727723982

H -2.53566451698454 -0.97627008721177 -4.57881495155505

H -4.53036511021823 0.49365001530583 -4.74776571127488

H -5.17763449026537 1.88837624440870 -2.79243922102345

Transition state of formation of m/z 271 (CO_2_ loss) from m/z 315 in nifedipine [M+H]^+^

C -2.03227853547959 2.15188794032161 -2.32720449687597

C -1.25499114418846 1.88306657461272 -1.19298292012855

C -1.38478730551238 0.71825634588922 -0.43119473327730

C -2.41784250085905 -0.14336015116008 -0.78012697075573

C -3.22934310560245 0.12418530356144 -1.88043975266024

C -3.03098594476598 1.26054005144232 -2.66261751757366

N -0.31069383625389 2.89407045774259 -0.83685189008069

O -0.47910055303210 3.48436137047386 0.48116572697296

C 0.32619948502298 2.75975611520510 1.37527604934849

C 0.70075764021956 1.46660105014950 0.74825943609494

C -0.37656073650367 0.41073154030302 0.69617598753228

C 1.96771291728805 1.22119473955693 0.29404731310658

N 2.24172131581571 -0.02553254754307 -0.17004825338921

C 1.47970254245338 -1.15777540579976 0.12351582471863

C 0.23727045322027 -0.98122771736758 0.63560914220648

C 2.21185050084558 -2.44333533734285 -0.12776409950428

C 3.06651535797511 2.23133919192907 0.21690705340341

O 0.25652834831144 3.58583640223251 -1.62336881794229

O 0.55474206258509 3.19007424779178 2.45103410340393

C -0.58283835171176 -2.03938355696978 1.27911490440951

O -1.67205807375799 -1.79783610277124 1.75480537258129

O -0.01237556442137 -3.24549150329004 1.31984266043017

C -0.76647541953983 -4.28636396815179 1.98744123317052

H -1.87857352716014 3.07200192945212 -2.87435070825821

H -2.57676467779638 -1.03977011422996 -0.19961968272333

H -4.01517936660916 -0.57422890621341 -2.13764276332211

H -3.66655705249428 1.46074229626462 -3.51462306772246

H -0.97233188551713 0.44614346435923 1.61730583854670

H 3.15582052377196 -0.18537057646295 -0.56841328474930

H 2.68040027105391 -2.80470606591807 0.79029692369984

H 2.99801117081435 -2.28914946886765 -0.86964312115404

H 1.54651740669960 -3.22224938365799 -0.48341106148177

H 3.96620757172137 1.85975211435950 0.71232290684297

H 2.78737180147988 3.17079535487921 0.68691728190874

H 3.32015744023023 2.43444576323994 -0.82819269179071

H -0.13686165978310 -5.16938029162129 1.94034488660522

H -1.71033431964343 -4.45547993917177 1.47243009294248

H -0.96039324887636 -4.00208121722702 3.01981309546471

Transition state of formation of m/z 283 (MeOH loss) from m/z 315 in nifedipine [M+H]^+^

C 1.15782807443350 1.40955270288414 0.67595075736491

C 2.43303310569857 1.10429181774475 0.14148048633977

N 2.78394191256689 -0.14278449105983 -0.16945456359548

C 2.03018330514742 -1.17738721446629 0.22141139249821

C 0.72808454599435 -1.00354831744106 0.75967151892595

C 0.04533075173942 0.35326741026345 0.66381559823189

C 3.47852696426733 2.14128676457725 -0.10267211385510

C 2.68447067537362 -2.51250768693015 0.08016814655601

C -0.83570200136027 0.52842015004919 -0.58500909610440

C -0.50727310285659 -0.12180826635628 -1.77615972790944

C -1.26614542361163 0.02577095650033 -2.93131577621228

C -2.41038775391610 0.81357961565040 -2.92427460674304

C -2.78751800392669 1.44963705080051 -1.75308985659642

C -1.99927001789133 1.31686026948856 -0.61516877972460

C 0.15437027674162 -2.04908248926281 1.45356771343426

C 0.96624109983114 2.65785193927842 1.15598020390743

O 0.51535675941461 -3.19436648232381 1.81405121716939

O -1.14610150187930 -1.96502861941765 2.13696457744231

C -2.41545094656080 -1.96605447714068 1.39002636805520

O 0.91312009641742 3.71964648200619 1.56435968717458

N -2.46871693878045 2.04576274013347 0.57720852995854

O -1.62095922677151 2.44738451981429 1.37561681714849

O -3.66213188218752 2.21524324461626 0.70876916495669

H -0.56454340708927 0.49552264484187 1.54978814305308

H 3.69654263969233 2.16008534579951 -1.17416971362026

H 4.40387537698095 1.84757345267187 0.39558019616555

H 3.19543142373334 3.14307647994032 0.21353562174705

H 3.52098949643026 -2.42171653638638 -0.60805613120113

H 1.99364088035679 -3.28855184212129 -0.24676094849514

H 3.07416448598635 -2.83469681893817 1.05140909255057

H 0.36171358040187 -0.76363617417333 -1.80816849323936

H -0.96216465722243 -0.48339128799271 -3.83665686917143

H -3.00735694464731 0.92932439554575 -3.81868654093972

H -3.68058480089663 2.05599756260446 -1.70434026708089

H -2.56079115507281 -0.98730172403174 0.94917929386420

H -3.17996080917881 -2.16717017123157 2.13421830230848

H -2.38753513651670 -2.74376946634600 0.62867645407157

H -0.67425174084161 -3.03733347959122 2.41255420156453

Nifedipine [M+Na]^+^

C -0.42988133861758 1.37206926995232 -0.38144960120335

C 0.78972261363398 0.98490173815104 -0.87100636809316

N 1.01928014897016 -0.35089476603107 -1.02899611497641

C 0.24012980990029 -1.35327970826309 -0.45863535390790

C -1.00321694266421 -1.03416457837392 -0.03882341746867

C -1.56186204753894 0.34957208676435 -0.30795385588675

C 1.95471789423700 1.85103530044766 -1.24662915620194

C 0.93470147963717 -2.67468870096017 -0.35935733458369

C -2.38877747255352 0.40581983365376 -1.60300799846875

C -1.91869465632741 -0.24713911041305 -2.74348011896917

C -2.53468885677319 -0.11965963376586 -3.98044190586381

C -3.65202719570254 0.69450165654785 -4.13417553920797

C -4.14795888123644 1.36804135261544 -3.03457709202189

C -3.53109767830772 1.19664488203947 -1.79677042427730

C -1.84564164558793 -2.01609293043722 0.67202246356093

C -0.72282474349453 2.70429203243943 0.09643265376507

O -1.49561552940891 -3.10627769166848 1.05977624557100

O -3.09569783202145 -1.54054518503568 0.85384194043442

C -4.00224558689749 -2.41371030679532 1.54907478906287

O -1.80304581055919 3.02129644490498 0.60816040612978

O 0.26626145320052 3.59417891935145 -0.02057007926746

C 0.01601516840487 4.93865609117051 0.42029669409630

N -4.13858619128190 1.93104131838896 -0.70016208359488

O -4.27287498126988 1.39279608822833 0.39602747301815

O -4.48659934716695 3.09727338088468 -0.89681384145194

H 1.92397093496076 -0.62515369381863 -1.37690126944462

H -2.20713932977479 0.61096131838535 0.51875943983141

H 1.64064305438851 2.70563288098566 -1.83898176748207

H 2.68064131181308 1.27395245431660 -1.82063035548002

H 2.45376824144132 2.23667506711603 -0.35685601515148

H 1.66446167380116 -2.77714280578257 -1.16642344913288

H 0.23280907403726 -3.50015466542078 -0.39498283405860

H 1.46907769493756 -2.75045238875644 0.59071826711841

H -1.04255788779620 -0.87174875579074 -2.65624768330453

H -2.13648242780226 -0.65696558495626 -4.83128046935230

H -4.13401744119267 0.79714854318080 -5.09625672238512

H -5.01651749865838 2.00589502021411 -3.11079784859582

H -4.93430153398251 -1.86173617811616 1.62520965385996

H -3.61244710188189 -2.65634062208246 2.53670964069057

H -4.14422238196859 -3.33589149605702 0.98757990817964

H -0.16016453021761 4.95920107442004 1.49509477610363

H -0.83861639242224 5.35994912854730 -0.10715015633023

H 0.91812627945865 5.49205642207105 0.17768635340287

Na -3.75881356971501 3.47551649774765 1.39128815133787

m-Nifedipine [M+Na]^+^

C 1.83645716640854 -0.36741431806524 -1.56461283900908

C 0.70179673115596 -1.17268636622333 -1.56113534312355

C -0.08796565913861 -1.31014062035529 -0.41316935077598

C 0.29776377311775 -0.61240543585263 0.72023186009526

C 1.39712811118780 0.23412572022593 0.68691454991122

C 2.19710618814581 0.36268359109815 -0.43875210153950

C -1.38351304581092 -2.14229258757131 -0.40979323429073

C -2.10426370405250 -1.96975814595116 0.91493265682880

C -1.98191395332607 -2.90624614801754 1.88686893379038

N -1.30777325484271 -4.06704290901573 1.59999681683602

C -1.02011809974286 -4.49387776910889 0.31189892396133

C -1.14359047349494 -3.61048629225819 -0.70668317151497

C -2.55894810140463 -2.85641131392057 3.26768644595102

C -0.66186019902411 -5.94416087262398 0.21103429971140

C -1.09224788844574 -4.08533727257493 -2.09815131067831

O -0.79997357311949 -5.20223638185568 -2.45820462786585

O -1.44565707439246 -3.11153438532369 -2.97394717031956

C -2.71311864863174 -0.67172408413850 1.17470075787913

O -2.63142198423677 -0.05823800784340 2.24510489332452

O -3.30475659238049 -0.14121360249895 0.11134871882548

C -3.84769710779296 1.18655680174857 0.23150391586323

C -1.49474249907694 -3.51276546306861 -4.35525811825469

N 1.66597524011243 1.05829400704095 1.86153100434822

O 2.70256870887797 1.66915894935146 1.94003210509933

O 0.78080092043859 1.11399561177536 2.74171454691890

H 2.43102054743809 -0.28469156690000 -2.46411581487297

H 0.41691749125682 -1.69518807000038 -2.46314865633090

H -0.24966377147927 -0.72013962697802 1.63775638575672

H 3.04948379021715 1.02542326995585 -0.43670350289643

H -2.00454876224161 -1.73330425708483 -1.20448670156333

H -1.28841879983600 -4.77438181728689 2.31702160459888

H -3.22827712262448 -2.01437145708036 3.39860147371327

H -1.75932002977817 -2.78166013808872 4.01076971145386

H -3.10868360299680 -3.77699566592197 3.47535897886239

H -0.47560272311951 -6.36038794266722 1.20272133189283

H 0.21656470514733 -6.09026652984552 -0.41082451859498

H -1.46869888842372 -6.50858875200450 -0.25875506713834

H -4.30203004019909 1.39894446492624 -0.73071963001658

H -3.05079617951292 1.90134022276169 0.43678491675842

H -4.59281522235638 1.22120819339462 1.02400188929057

H -1.78804281863788 -2.62362976187536 -4.90573473340597

H -2.22669180501390 -4.30742279912227 -4.49074555879512

H -0.51925033825351 -3.86892285030470 -4.68271007596889

Na -1.27984141011694 1.09954237914973 3.47047480528451

p-Nifedipine [M+Na]^+^

C 0.28752980036800 0.52626259350968 -1.47797461535977

C -0.50143295486094 -0.61321905262693 -1.47878551414153

C -0.46824008398451 -1.49966901874723 -0.39870305758938

C 0.52331001814365 -1.35089133497857 0.56696082089383

C 1.35019677795438 -0.23266834902320 0.57474081433682

C 1.15480815981252 0.72398935345061 -0.40717634978345

C -1.59532413971652 -2.50797460728423 -0.19958690224409

N 1.65654919419695 2.08322700364382 -0.16078669867417

C -2.18686166183481 -2.36321171051286 1.20506287554671

C -2.06739895965052 -3.36604097506772 2.12625911768799

N -1.44911509968318 -4.52745698170114 1.76779287509512

C -1.05064855409632 -4.85959129321189 0.48155595439391

C -1.15191808305771 -3.93132158918153 -0.49407564039083

C -0.56115023184746 -6.26914910496637 0.33917905904414

C -2.53629807235721 -3.35775184234825 3.55263760158665

C -2.80063327990920 -1.08094602230408 1.50692909416148

C -0.82429299773524 -4.29791153615214 -1.88321720522449

O -0.28029746459359 -5.31855093909870 -2.23443128477651

O -1.22448207786266 -3.34741614511878 -2.76235234924062

C -0.96406741679785 -3.64810047429024 -4.14675921491861

O -3.58564290849121 -1.05083944233371 2.58754000737688

O -2.63062068848813 -0.06369163057608 0.82337103864443

C -4.22133341523260 0.19750952411942 2.91504808445350

O 2.83579327355139 2.30930621667766 -0.09887932876863

O 0.75398632642382 2.91929511916697 0.02486427421711

H 0.21000809023020 1.26699532558359 -2.26428637641047

H -1.21141877447445 -0.77718798165189 -2.27752875127116

H 0.59516118405656 -2.06378716271639 1.37780812043473

H 2.06659716295950 -0.06456979962255 1.36770535496136

H -2.36742474915003 -2.23706389593648 -0.92167903663829

H -1.37564591157528 -5.24822379561801 2.46607308845859

H -0.67612212639068 -6.81198849237572 1.27860061787350

H 0.48820636109000 -6.28637611142099 0.04757532679542

H -1.10221367284410 -6.79661859721655 -0.44356834413021

H -2.26176049794392 -2.43793232666305 4.06167006627644

H -2.10350810924867 -4.19670559460055 4.09733120984319

H -3.62200449276931 -3.44050952659708 3.59840031987432

H -1.36666245519011 -2.81047679099866 -4.70901406250458

H -1.45888180722334 -4.57471775622402 -4.43190355654172

H 0.10679021323025 -3.74993120229949 -4.31633102999185

H -3.47536475629895 0.95458484437655 3.15785970126508

H -4.83503680756453 -0.01231467226744 3.78532821638439

H -4.83851852403130 0.53841614780636 2.08576475022656

Na -1.43949578711281 1.72932962739799 0.35586092876821

Nifedipine [M+Na]^+^, after two protons are transferred

C -0.56851389734516 1.03203196996115 -0.15983706065813

C -1.32151578026385 -0.15136409223913 -0.07366088493301

N -0.75881586082391 -1.30085388738339 0.29784543672556

C 0.53616774687793 -1.35124208904075 0.60732834330335

C 1.34190411932371 -0.20643661796867 0.56005611695915

C 0.79264282412720 1.01018235847360 0.17260745527957

C -2.78431918155040 -0.21421235398709 -0.40765986612579

C 1.09664528025583 -2.68085295861668 1.01287634253528

C 1.57279857104447 2.28301544528395 0.25356140846592

C 1.20682562510926 3.13737872267936 1.30256839238714

C 1.84970921121705 4.34222789036133 1.52856777293307

C 2.89727532156179 4.72470982823332 0.69977514361438

C 3.28224603043125 3.90355493250841 -0.34462795609158

C 2.62433132949790 2.69418577558366 -0.57980151964791

C 2.79640680382020 -0.30767655330083 0.89319433572368

C -1.16847749030909 2.27507651269860 -0.71016542943278

O 3.29998592635638 -0.09695775761886 1.95780407355959

O 3.49085956774409 -0.68968782345827 -0.21271864502517

C 4.92256605833978 -0.82298395564595 -0.05132745873454

O -0.71263784386080 2.86954480423338 -1.68211514212456

O -2.25449216994669 2.66570258075240 -0.07704130581621

C -2.95193252046928 3.82546990806216 -0.59128439017753

N 3.14728537840038 1.89654901655218 -1.67052846856064

O 2.19537595719458 0.92552769661063 -2.08887195302910

O 3.26113774364211 2.76096413290725 -2.80802244486160

H -3.02673097437338 0.33933478842160 -1.31600295472679

H -3.37824365493945 0.21456781696853 0.40170081316541

H -3.07165813971096 -1.25484707265366 -0.53369810645080

H 0.29385697389721 -3.40972316410742 1.08234520454941

H 1.60695510562659 -2.61355781019603 1.97567193297555

H 1.82749091555215 -3.03129052703966 0.27931424507922

H 0.40592739493010 2.82432936144975 1.95921214826122

H 1.54375331681486 4.97137452602717 2.35311264180085

H 3.41705307212611 5.65869652496410 0.86415786268261

H 4.09303455218705 4.19605756322021 -0.99469486526864

H 5.14011633489883 -1.55984714328973 0.71847233941947

H 5.35313668347440 0.13688606197969 0.22637627018635

H 5.29521672195941 -1.15298254416315 -1.01637560389864

H -3.78397752329427 3.97895204072921 0.08744515603856

H -3.30729693955329 3.62395919278114 -1.60013316921888

H -2.28697174937185 4.68636734487960 -0.59511019211081

H 2.58755533147439 0.08544024862549 -1.78456199074722

H 4.04682078350491 2.43985145994135 -3.27268079218277

Na 0.88366304442235 2.54324784581997 -3.16088323582219
